# Can exercise combined with transcranial direct current stimulation improve cognitive function in older adults? A systematic review and meta-analysis

**DOI:** 10.3389/fnins.2026.1836124

**Published:** 2026-05-08

**Authors:** Shengfei Hu, Yi Yang, Xinshu Li

**Affiliations:** 1 South-Central University for Nationalities, Wuhan, China; 2 Southwest University, Chongqing, China; 3 Zhongnan University of Economics and Law, Wuhan, China

**Keywords:** cognitive function, cognitive impairment, executive function, exercise, memory, older adults, transcranial direct current stimulation

## Abstract

**Objective:**

This study investigated whether combining exercise with transcranial direct current stimulation (tDCS) improves overall cognition, memory, and executive function in older adults.

**Methods:**

Following PRISMA guidelines, we systematically searched databases including PubMed, Web of Science, CNKI, and Wan Fang for randomized controlled trials (RCTs) examining the combined effect of exercise and tDCS on cognitive function in older adults. Used RStudio (version 4.2.0) to merge effect sizes and represent them as SMD with a 95% confidence interval (CI). The main effects are synthesized using a random effects model, and heterogeneity sources are explored through subgroup regression and sensitivity analysis.

**Results:**

The combined exercise and tDCS intervention significantly improved global cognitive function in older adults (SMD = 0.62, 95% CI: 0.36 to 0.89, *p* < 0.0001). Significant enhancements were observed in executive function (SMD = 0.54, 95% CI: 0.16 to 0.92, *p* = 0.005) and general cognitive ability (SMD = 0.75, 95% CI: 0.21 to 1.30, *p* = 0.006), while memory showed a non-significant improvement (SMD = 0.58, 95% CI: −0.03 to 1.19, *p* = 0.063). Both interventions lasting less than 6 weeks (SMD = 0.94, 95% CI: 0.60 to 1.27, *p* < 0.0001) and those lasting 6 weeks or longer (SMD = 0.24, 95% CI: 0.10 to 0.37, *p* = 0.0006) positively impacted cognitive function. However, the effect size was larger for cognitively healthy older adults (SMD = 0.69, 95% CI: 0.20 to 1.18, *p* = 0.006) compared to those with cognitive impairment (SMD = 0.60, 95% CI: 0.29 to 0.92, *p* = 0.0002). The combination of tDCS and integrated exercise produced the largest effect size (SMD = 1.74), despite high heterogeneity, while the combination of tDCS and Tai Chi produced the smallest but most robust effect (SMD = 0.25, I ^2^ = 0%), indicating that exercise type significantly regulates the intervention effect of tDCS (*p* = 0.0015). Regression analysis shows that tDCS stimulation time has a significant positive regulatory effect on cognitive function in elderly people (*p* = 0.0002), while the combined intervention period (*p* = 0.030) and single exercise time (*p* = 0.034) both have a significant negative regulatory effect.

**Conclusion:**

Based on limited evidence, we found that a combined intervention of exercise and tDCS is a potentially effective means of improving cognitive function in older adults. However, the extent of improvement varies with the cognitive domain, baseline performance level, and intervention plan.

## 1 Introduction

According to data from the World Health Organization (WHO), the global population aged 60 years and over reached one billion in 2019. It is projected that this demographic will grow further, reaching 1.4 billion by 2030 and potentially 2.1 billion by 2050, due to ongoing population aging (World Health Organization, 2021). However, the onset of neurodegenerative diseases in older adults due to ageing represents a significant challenge within ageing populations. Ageing is associated with a decline in cognitive function, including attention, memory, language, and executive function (Celsis, 2000). This decline can progress to mild cognitive impairment and even dementia, severely affecting daily functioning and health status (Syed et al., 2025). According to the WHO 2021, the number of people with dementia is estimated to reach 82 million by 2030 and 152 million by 2050 (World Health Organization, 2021). Consequently, as life expectancy increases, strategies to delay age-related cognitive decline are receiving increasing attention. Furthermore, optimal cognitive function is fundamental for human survival, development, and healthy ageing. While there is currently no cure for dementia, slowing cognitive decline remains a key focus for prevention. Notably, cognitive impairment is influenced by multiple factors, with age and educational attainment being primary determinants (Yi et al., 2022). Unhealthy lifestyle behaviours and insufficient physical activity can also adversely impact brain function and structure, leading to cognitive impairment (Dhahbi et al., 2025).

Non-pharmacological interventions have been shown to enhance cognitive function in older adults (Yang et al., 2022; Yang et al., 2023; Giordano et al., 2017; Hortobágyi et al., 2022). Common interventions include physical exercise, cognitive behavioral therapy, and non-invasive neurostimulation techniques (Feng et al., 2025; Vasquez-Carrasco et al., 2025). Among these, physical exercise serves as a crucial regulator of healthspan and an effective strategy for slowing cognitive decline in this population (Yi et al., 2022; Yang et al., 2022; Baranowski et al., 2020; Falck et al., 2019). Research indicates that aerobic exercise can effectively enhance cognitive agility, memory, executive function, and mood. These cognitive benefits are associated with structural and functional changes in the brain, including increased hippocampal volume and elevated levels of brain-derived neurotrophic factor (BDNF) (Yang et al., 2023). Exercise induces neuroplasticity and neuroprotection, mechanisms activated during cognitive processes. Furthermore, both acute and chronic moderate-to-high-intensity resistance training can improve information processing speed and attention in older adults (Dhahbi et al., 2025). In recent years, non-invasive neurostimulation techniques have become widely used in cognitive neuroscience research. By targeting electrochemical processes within the central, peripheral, and autonomic nervous systems, these techniques facilitate neural functional remodelling and the reversal of pathological states. Transcranial direct current stimulation (tDCS), known for its convenience, non-invasiveness, and efficiency, is particularly popular. Regulating cortical excitability and neural plasticity is considered an important mechanism for enhancing cognitive and clinical abilities in neurodegenerative diseases (Pellicciari and Miniussi, 2018).

tDCS is a non-invasive neural modulation technique involving the application of a weak direct current (0.5–2 mA) through the scalp to modulate neuronal membrane potentials. Cathodal stimulation reduces cortical excitability by hyperpolarising neurons, while anodal stimulation increases excitability via subthreshold depolarisation (Giordano et al., 2017). Research indicates that tDCS can enhance visual, motor, sensory, and memory functions, potentially alleviating cognitive impairments associated with both physiological and pathological ageing (Dedoncker et al., 2016; Impey et al., 2017). These effects are primarily mediated by modulating excitability within cortical regions such as the primary motor cortex, dorsolateral prefrontal cortex, posterior parietal cortex, inferior frontal cortex, and their interconnected networks (Polania et al., 2018). Consequently, tDCS holds significant therapeutic promise for promoting neural synaptic reorganisation and has been investigated for treating various neurodegenerative diseases and mental health conditions.

It is worth noting that a potential strategy involves combining non-invasive brain stimulation techniques with motor training to enhance neuroplasticity and neuroprotection, thereby improving cognitive function (Indahlastari et al., 2021; Koch et al., 2020; Steinberg et al., 2019; Moreau et al., 2015). However, research into the effects of combining physical exercise and tDCS on cognitive function in the elderly is limited. Studies indicate that combining aerobic exercise with tDCS can significantly improve cognitive function across multiple domains more effectively than single interventions, suggesting its potential value in enhancing cognitive performance in complex occupational settings (Ward et al., 2017). Aerobic exercise and tDCS appear to be effective strategies for slowing cognitive decline in cases of mild cognitive impairment and dementia (Talar et al., 2022). However, only one of the included studies employed a combined aerobic exercise and tDCS intervention. This paper will therefore use a meta-analysis to compile the latest research evidence on the effects of combining physical exercise and tDCS to improve cognitive function in elderly people. This study aims to explore the benefits of combining physical exercise with tDCS for cognitive function in the elderly and to provide recommendations for treating cognitive decline in this population.

## 2 Methods

This study strictly adhered to the PRISMA 2020 guidelines for literature screening, inclusion, data processing, and analysis (Page et al., 2021). It was registered with the PROSPERO system (registration number CRD420251059150).

### 2.1 Searching strategy

Two researchers utilized the PubMed, Web of Science, Wan Fang, and China National Knowledge Infrastructure (CNKI) databases to retrieve Chinese subject terms based on the PICOS principle. These terms pertained to physical activity, exercise intervention, tDCS, and cognitive function domains, including executive function, working memory, cognitive flexibility, and inhibitory control. They employed a ‘subject term + free term’ search strategy. The literature search encompassed all publications up to June 1, 2025.

### 2.2 Inclusion and exclusion criteria

This study employed the PICOS framework to establish literature screening, inclusion, and exclusion criteria. Inclusion criteria: (1) Participants: Older adults aged ≥60 years, consistent with the World Health Organization (WHO) definition (Amuthavalli et al., 2022). (2) Intervention: The experimental group received a combined intervention of exercise and tDCS. Exercise was prescribed according to ACSM guidelines and included activities such as sports, games, exercise training, and fitness activities (Thompson, 2023). (3) Comparison: The control group received either exercise alone, tDCS, or no intervention. (4) Outcomes: Primary outcomes included cognitive function, executive function, working memory, cognitive flexibility, and inhibitory control. (5) Study design: Randomised controlled trials (RCTs) and randomised crossover trials. Exclusion criteria: (1) Literature not published in English or Chinese. (2) Non-experimental study designs. (3) Studies where the control group lacked pre- and post-test assessments. (4) Studies unrelated to exercise prescriptions, tDCS intervention, or cognitive function. (5) Reviews, theses, conference papers, qualitative studies, other secondary literature, or studies with incomplete or non-extractable original data.

### 2.3 Literature screening

NoteExpress software was utilized to remove duplicate records from the retrieved literature. Two researchers independently screened the literature based on predefined inclusion and exclusion criteria. Initially, they screened titles and abstracts, followed by a full-text review of potentially eligible studies. Consensus between the two researchers was required; if disagreements persisted after discussion, a third researcher was consulted for adjudication. Following the literature selection, the third researcher performed cross-referencing and organization of the database.

### 2.4 Evaluation of literature quality

The risk of bias for the included studies was independently assessed by two reviewers using the Cochrane Risk of Bias 2.0 (ROB2) tool (Cumpston et al., 2019), which evaluates bias across the following domains: bias arising from the randomization process, bias due to deviations from intended interventions, bias due to missing outcome data, bias in measurement of the outcome, and bias in selection of the reported result. Each domain was rated as “low risk,” “some concerns,” or “high risk.” Any disagreements were resolved through discussion with a third reviewer.

### 2.5 Evidence level assessment

Two researchers independently assessed evidence grades using the Grading of Recommendations, Assessment, Development and Evaluation (GRADE) system (Guyatt et al., 2008), which categorizes evidence into four levels: “very low,” “low,” “moderate,” and “high.” The assessment criteria included the following: heterogeneity, where moderate heterogeneity (I^2^ > 25%) warranted a one-level downgrade, and high heterogeneity (I^2^ > 75%) warranted a two-level downgrade; precision, which was downgraded by one level if no statistically significant difference was observed; and publication bias risk, which was downgraded by one level if Egger’s test yielded a *p*-value < 0.05.

### 2.6 Statistical analysis

Statistical analyses and graphical presentations were conducted using the “meta,” “metafor,” and “ggplot2” packages in R (version 4.4.3). A random-effects model with the DerSimonian–Laird estimator was employed to synthesize main effects using inverse variance weighting. The Jackson method was used to estimate *τ*^2^ and τ and to calculate their confidence intervals (Oliveira, 2013). Effect sizes were derived using a predefined formula based on differences in means and standard deviations. Due to inconsistent units of measurement across studies, the standardized mean difference (SMD) was adopted as the common effect size metric, with Hedges’ g used for small-sample correction (Lifeng Lin and Aloe, 2021). Effect sizes were interpreted using Cohen’s benchmarks: small (≈0.2), moderate (≈0.5), and large (≥0.8) (Muller, 1989). Heterogeneity was quantified using the I^2^ statistic, with values of 25, 50, and 75% indicating low, moderate, and high heterogeneity, respectively (Nakagawa et al., 2017). To provide a comprehensive characterization of between-study variability and to illustrate the range of plausible effects for future research, we calculated 95% prediction intervals (PIs) alongside the 95% confidence intervals (CIs) for the pooled estimates, and statistical significance was defined as *p* = 0.05. Finally, sensitivity analyses using a leave-one-out approach were conducted to assess the robustness of the findings (Wolfgang Viechtbauer and Cheung, 2010).

## 3 Results

### 3.1 Literature search results

**Figure 1:**
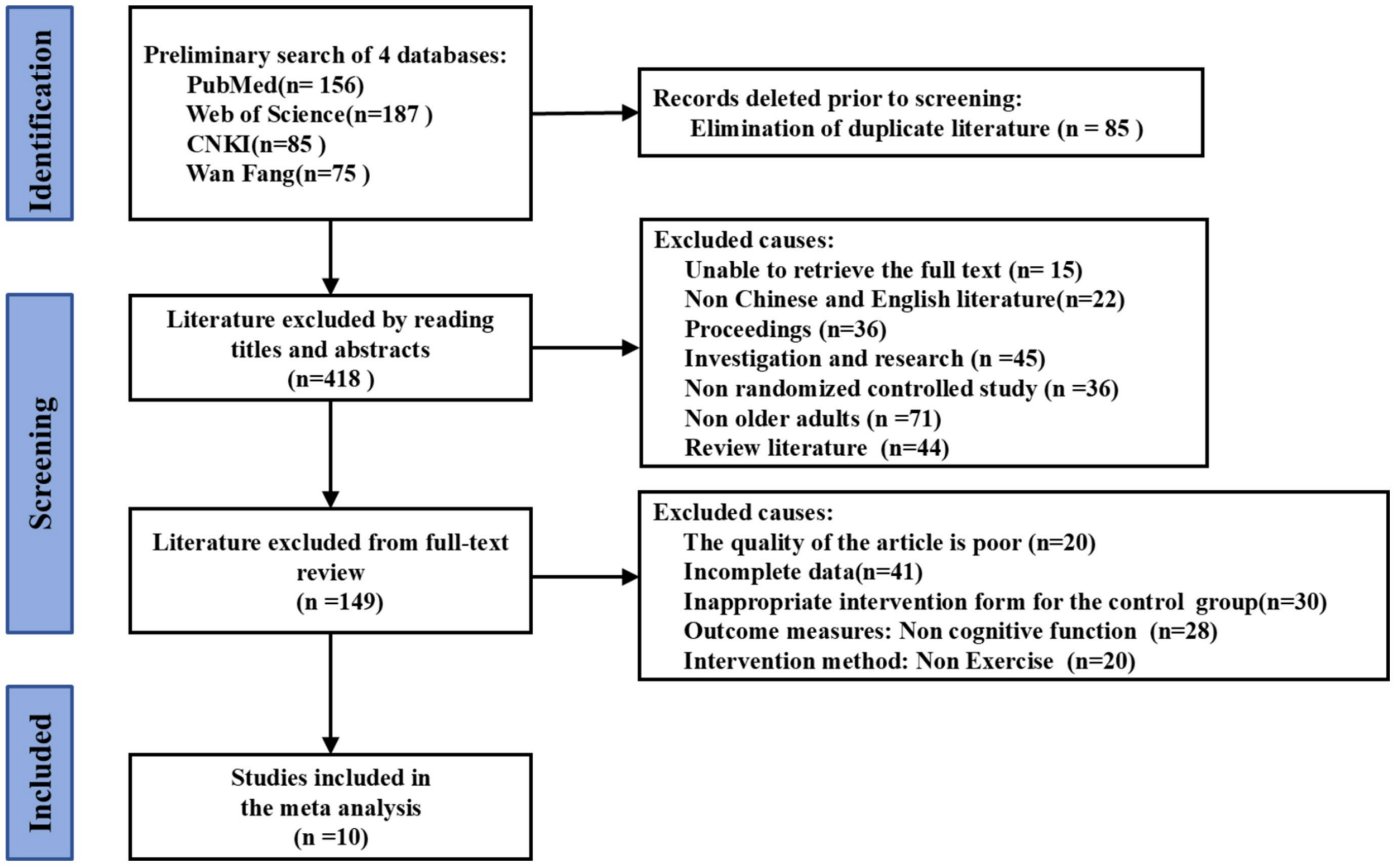
Literature retrieval and screening process.

The literature screening process followed a standardized protocol. After deduplicating search results using NoteExpress software, 503 articles remained (160 in Chinese, 343 in English). Two researchers independently screened records in two phases: an initial title/abstract assessment identified potentially eligible articles for full-text review. Disagreements were resolved through third-reviewer adjudication and group consensus to ensure database integrity. Following the exclusion of 85 duplicates, tiered screening of the remaining 418 records commenced. In the title/abstract phase, 269 records were excluded. Full-text evaluation eliminated 139 additional records. Ultimately, 10 studies met the eligibility criteria (Figure 1).

### 3.2 Inclusion of literature characteristics

The included studies were coded in Excel. Extracted characteristics comprised authors, publication year, nationality, sample details (e.g., age, sample size), physical activity intervention parameters (e.g., method, cycle duration, session duration, frequency), and tDCS protocols (e.g., stimulation parameters, location). Results are presented in Table 1.

**Table 1:** Characteristics of the included studies.

Study details	Country	Age\n\Gender	Intervention cycle	Intervention model	Exercise programme	Tdc parameters	Stimulation site	Cognitive measure	Cognitive effect	Research type
Schneider et al. (2021)	Isreal	73.9 ± 5.2; 25(Older Adults); Female(20), Male (5)	#	TDCS+walking	20 min walking	0.08 mA; 20 min	DLPFC	SCWT Test	Global cognitive function⬆	RCT
Longfei et al. (2025)	China	58.45 ± 2.82; 75 (Stroke patients); Female (26), Male (49)	4 weeks	TDCS+Comprehensive exercise	30 min\sessions,5sessions\week, Comprehensive exercise	2.0 mA, 30 min\sessions, 5 times\week	DLPFC	WCST Test; WMS-IV Test; MoCA Test	Global cognitive function⬆ Executive function⬆ Memory⬆	RCT
Ying et al. (2024)	China	60.74 ± 5.35; 79(MCI); Female (26), Male(49)	12 weeks	TDCS + Tai Chi	60 min\sessions,3sessions\week, Tai Chi	2.0 mA, 20 min\sessions, 3 times\week,	DLPFC, Upper left eye socket	MoCA Test; WMS-IV Test	Global cognitive function⬆ Memory⬆	RCT
Orcioli-Silva et al. (2021)	Italy	69.00 ± 6.74; 18 (Older Adults); Female (8), Male (10)	#	TDCS + walking	20 min\sessions, 5 sessions\week,	0.6 mA, 20 min\sessions, 5 times\week,	Left PFC, motor cortex (M1)	Digit Vigilance Test	Global cognitive function⬆	RCT
Conceição et al. (2021)	Brazil	70.80 ± 7.87; 20 (Parkinson’s Elderly) Female(10), Male(10)	#	TDCS + cycling	30 min, recumbent bicycle	2.0 mA, 20 min	DLPFC	TMT Test; MoCA Test	Global cognitive function⬆	RCT
Xu et al. (2023)	China	58–63; 180 (MCI); Female (111), Male (69)	12 weeks	TDCS + walking TDCS + Tai Chi	60 min\sessions, 3 sessions\week, Tai Chi or walking	2.0 mA, 20 min\sessions, 3 sessions\week	Right LPFC, upper left eye socket	MoCA Test; Stroop Test;MQ Test;	Cognitive function⬆Executive function⬆ Memory⬆	RCT
Manenti et al. (2016)	Italy	69 ± 9.1; 20(Parkinson’s Elderly) Female(10), Male(10)	2 weeks	TDCS+Comprehensive exercise	25 min\sessions, 5 sessions\week	2.0 mA, 25 min\sessions, 5 sessions\week	DLPFC	PD-CRS; Digit span; Frontal Assessment Battery;	Global cognitive function⬆, Executive function⬆, Memory⬆	RCT
Clark et al. (2021)	America	69 ± 9.1; 18(Older Adults); Female (13), Male (5)	6 weeks	TDCS + walking	30 min\sessions, 3 sessions\week	2.0 mA, 20 min\sessions, 3 sessions\week	DLPFC,	Nih Examiner; TMT Test	Global cognitive function⬆	RCT
Liao et al. (2021)	China	72.85 ± 4.35; 20(MCI); Female (13), Male (7)	12 weeks	TDCS + Tai Chi	40 min\sessions, 3 sessions\week	2.0 mA, 20 min/session, 3 sessions\week,	left DLPFC	MoCA Test; TMT Test; SCWT Test; CVVLT Test	Global cognitive function⬆, Executive function⬆, Memory⬆	RCT
Zhang et al. (2024)	China	56.92 ± 6.70 90 (older adults) Female (42), Male (48)	4 weeks	TDCS+walking	20 min\sessions, 5 sessions\week, 55–65% HRmax	2.0 mA, 20 min\sessions, 3 sessions\week		MoCA Test LOTCA Test	Global cognitive function⬆, Executive function⬆, Memory⬆	RCT

N: total sample size; #: indicates a one-time campaign; DLPFC, dorsolateral prefrontal cortex; MCI, mild cognitive impairment; HRmax, Maximum heart rate; PD, Parkinson’s disease; MoCA, Montreal Cognitive Assessment; MQ, Memory Quotient; RCT, Randomised controlled trial; ⬆, Significantly improved; PD-CRS, Unified Parkinson Disease Rating Scale; TMT, Trail Making Test; LOTCA, Loewenstein Occupational Therapy Cognitive Assessment; TMT, Trail Making Test; SCWT, Stroop Color-Word Test; CVVLT, Chinese Verbal Learning Test.

All ten included studies were randomised controlled trials (RCTs) incorporating pre- and post-intervention measurements. The pooled sample size across studies was 1,769 participants (range: 20–240 per study), all aged 60 years or older. Studies were categorised based on intervention duration: short-term exercise (<6 weeks) or long-term exercise (≥6 weeks). Six studies employed short-term interventions (Schneider et al., 2021; Longfei et al., 2025; Orcioli-Silva et al., 2021; Conceição et al., 2021; Manenti et al., 2016; Zhang et al., 2024), while four implemented long-term interventions (Ying et al., 2024; Xu et al., 2023; Clark et al., 2021; Liao et al., 2021). Studies were stratified based on the presence or absence of cognitive impairment into two groups: a cognitive impairment group and a non-cognitive impairment group. The cognitive impairment group comprised six studies (Longfei et al., 2025; Ying et al., 2024; Conceição et al., 2021; Xu et al., 2023; Manenti et al., 2016; Liao et al., 2021), while the non-cognitive impairment group included four studies (Schneider et al., 2021; Orcioli-Silva et al., 2021; Clark et al., 2021; Zhang et al., 2024). From the perspective of cognitive function domains, studies were categorized as assessing global cognitive function, executive function, or memory. Six studies evaluated global cognitive function (Longfei et al., 2025; Ying et al., 2024; Xu et al., 2023; Manenti et al., 2016; Liao et al., 2021; Zhang et al., 2024), six focused on memory (Longfei et al., 2025; Ying et al., 2024; Xu et al., 2023; Manenti et al., 2016; Clark et al., 2021; Liao et al., 2021), and seven examined executive function (Schneider et al., 2021; Longfei et al., 2025; Orcioli-Silva et al., 2021; Conceição et al., 2021; Xu et al., 2023; Manenti et al., 2016; Clark et al., 2021). From the perspective of intervention grouping of different exercise types combined with tDCS, two studies used tDCS+comprehensive exercise (Longfei et al., 2025; Manenti et al., 2016), four studies used tDCS+walking (Schneider et al., 2021; Orcioli-Silva et al., 2021; Clark et al., 2021; Zhang et al., 2024), three studies used tDCS+Tai chi (Ying et al., 2024; Xu et al., 2023; Liao et al., 2021), and one study used tDCS+cycling (Conceição et al., 2021). All studies employed established cognitive assessments. Consequently, the standardized mean difference (SMD) was selected as the effect size indicator.

### 3.3 Risk assessment of bias in included studies

**Figure 2:**
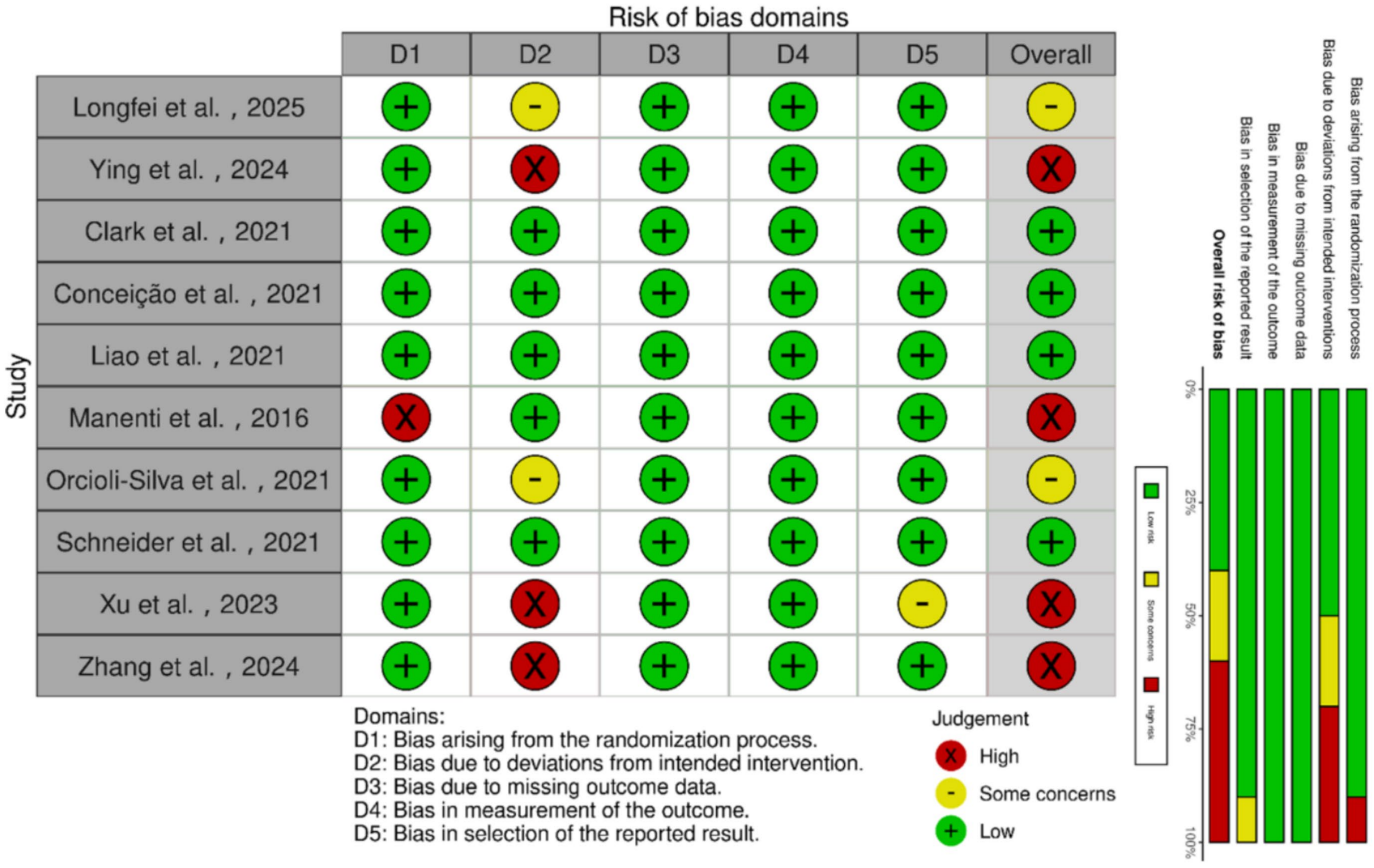
Risk assessment of bias in randomized controlled trials.

Assessment of the 10 included trials using RoB 2.0 (Figure 2) revealed an overall risk of bias categorized as ‘some concerns.’ Key methodological issues included inadequate randomization and allocation concealment, unclear blinding of outcome assessors, and potential selective reporting due to discrepancies between the registration protocol and the final published results. Furthermore, several studies demonstrated limitations in intervention implementation and adherence monitoring, attrition during follow-up, and handling of missing data, such as failing to clarify missing mechanisms or apply intention-to-treat analysis.

### 3.4 Meta-analysis of the effects of exercise combined with tDCS on cognitive function in older adults

**Figure 3:**
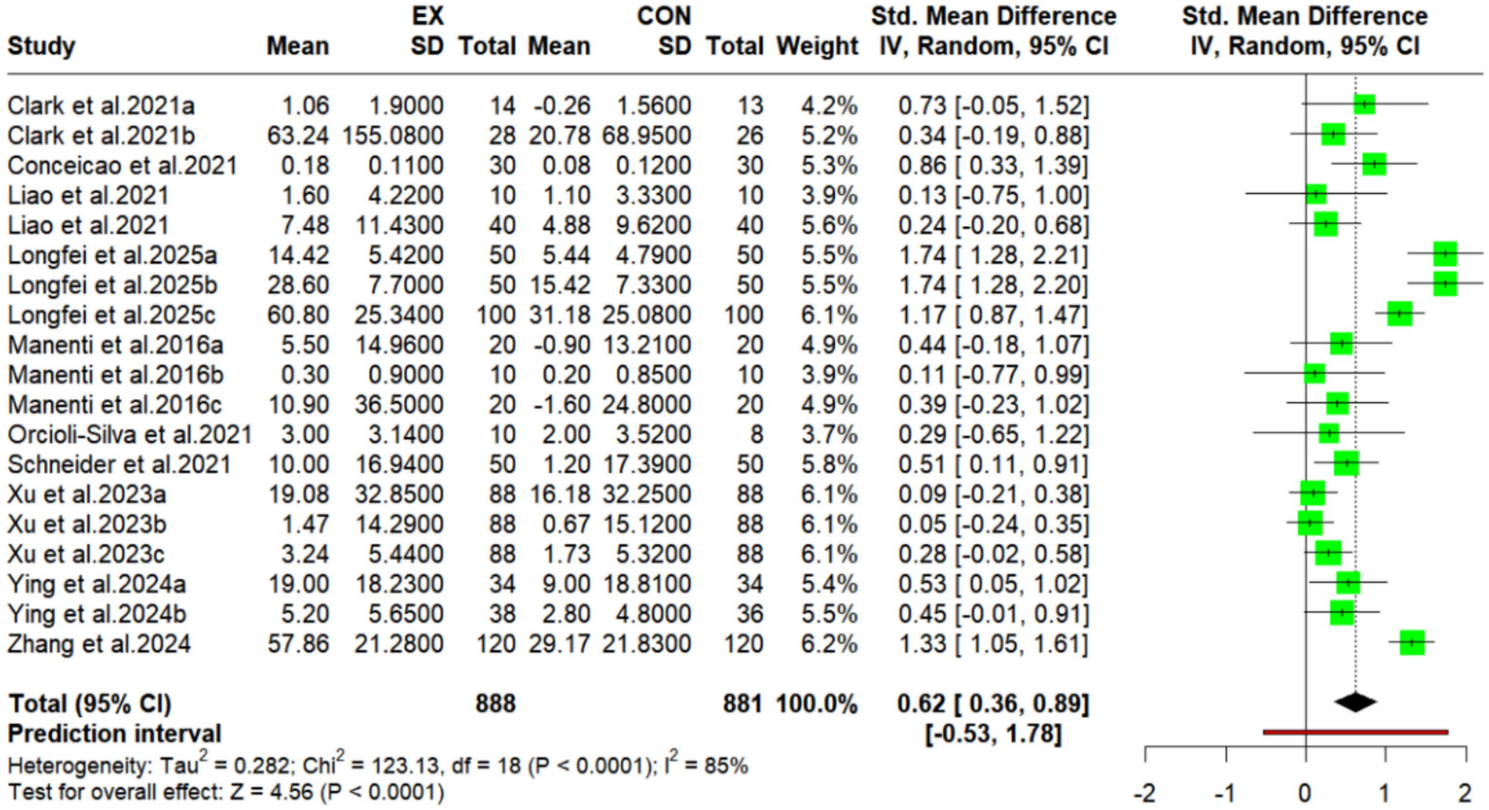
Meta-analysis forest plot of the effects of combined tDCS on cognitive.

This study incorporated data from 10 randomized controlled trials (RCTs), encompassing 19 distinct study outcomes and 1,769 participants, to evaluate the efficacy of combined exercise and tDCS interventions on cognitive function in older adults (Figure 3). Meta-analysis revealed a statistically significant improvement in cognitive function, evidenced by a standardized mean difference (SMD) of 0.62 (95% CI: 0.36 to 0.89; *p* < 0.00001). This SMD corresponds to a moderate effect size. Heterogeneity analysis indicated substantial between-study variability (I^2^ = 85%).

### 3.5 Results of subgroup analyses

#### 3.5.1 Subgroup analyses of the effects of exercise combined with tDCS on different cognitive dimensions in older adults

**Figure 4:**
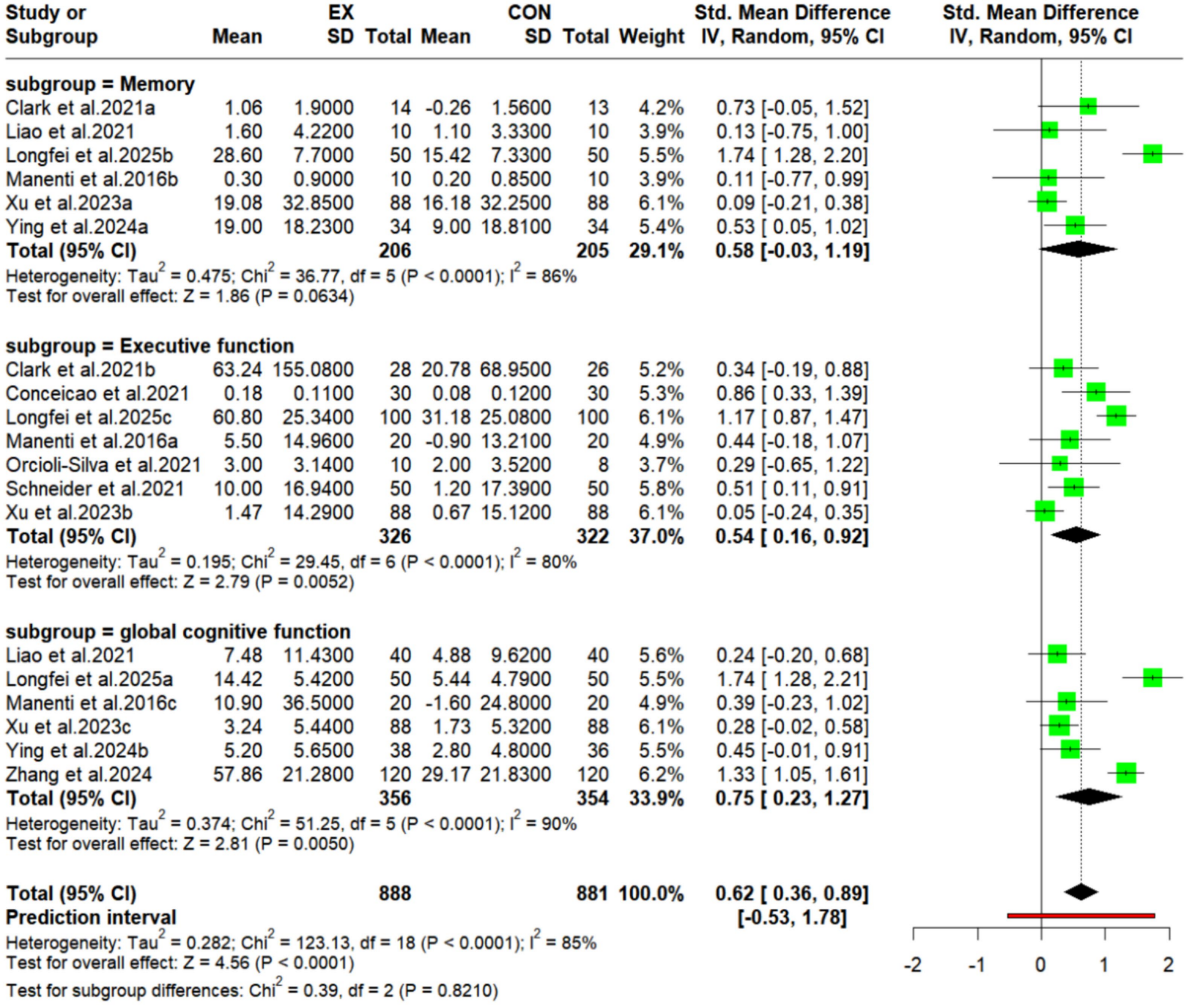
Subgroup analyses forest plot of the effects of motor-associated tDCS on different cognitive dimensions in older adults.

This study analyzed participants divided into three subgroups: executive function, memory, and global cognitive function(Figure 4). Results indicated the following: The combined effect size for the executive function subgroup (experimental group: *n* = 336; control group: *n* = 322) was SMD = 0.54 (95% CI: 0.16 to 0.92; *p* = 0.005), with high heterogeneity (I^2^ = 80%). The memory subgroup (experimental group: *n* = 236; control group: *n* = 235) had a pooled effect size of SMD = 0.58 (95% CI: −0.03 to 1.19; *p* = 0.063), also with high heterogeneity (I^2^ = 87%). The global cognitive function subgroup (experimental group: n = 326; control group: *n* = 324) demonstrated the largest effect size (SMD = 0.75; 95% CI: 0.23 to 1.27; *p* = 0.005), again with high heterogeneity (I^2^ = 89%). The high heterogeneity observed across all three subgroups suggests potential differences in intervention intensity, baseline sample characteristics (e.g., age, health status), or study design within these subgroups.

#### 3.5.2 Subgroup analyses of the effects of exercise combined with tDCS on cognitive function in older adults with different intervention cycles

**Figure 5:**
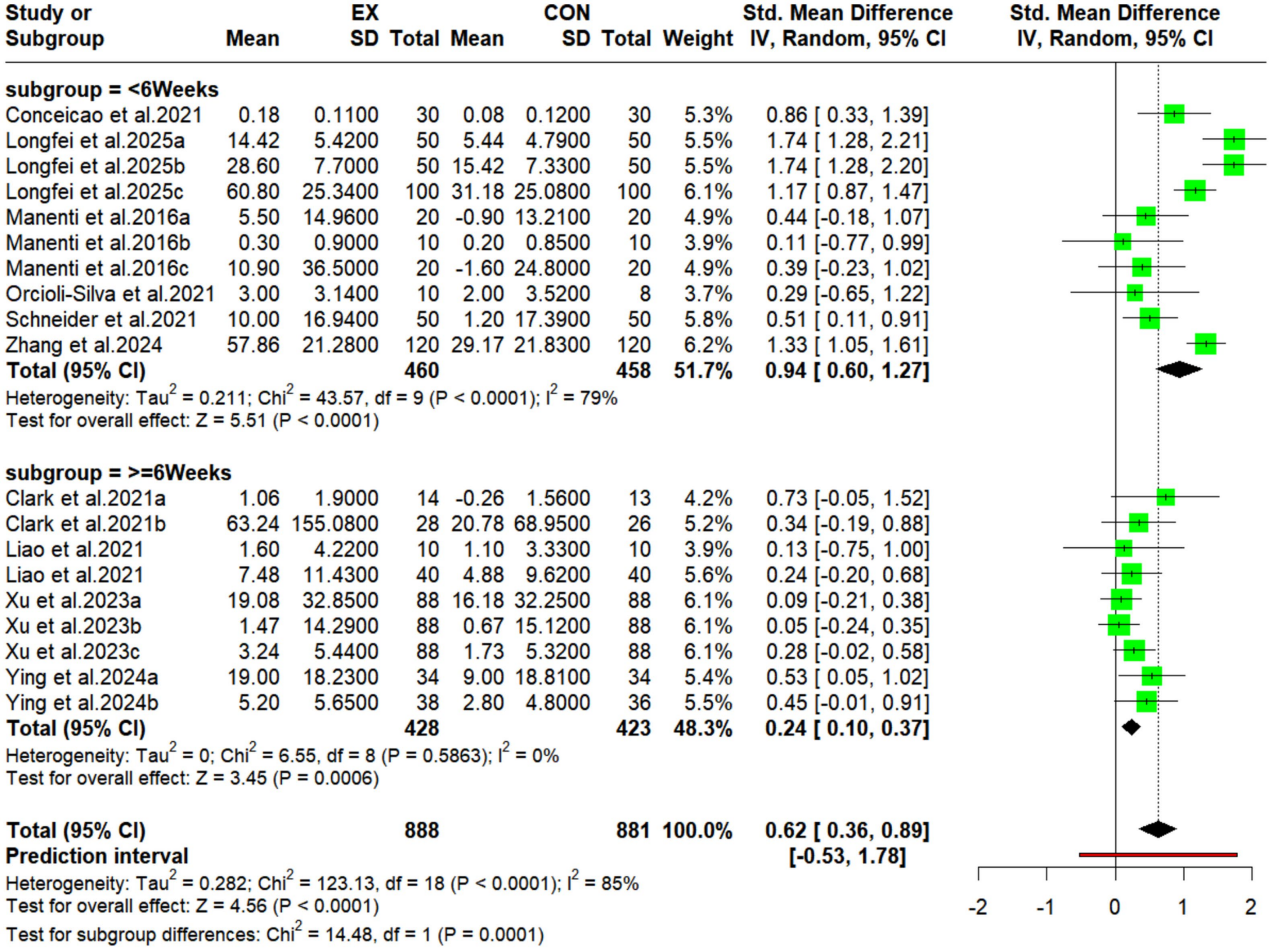
Subgroup analyses forest plot showing the effects of different intervention cycles of motor-associated tDCS on cognitive function in older adults.

This study stratified the analyzed intervention durations into two subgroups: <6 weeks and ≥6 weeks (Figure 5). Results demonstrated a significant pooled effect size for the <6 weeks subgroup (experimental group: *n* = 460; control group: *n* = 458; SMD = 0.94, 95% CI: 0.60 to 1.27; *p* < 0.0001), although heterogeneity was high (I^2^ = 79%). Conversely, the ≥6 weeks subgroup (experimental group: *n* = 428; control group: *n* = 423) yielded a smaller but statistically significant pooled effect size (SMD = 0.24, 95% CI: 0.10 to 0.37; *p* = 0.0006), with no observed heterogeneity (I^2^ = 0%). These results indicate that tDCS combined with exercise can effectively improve cognitive function in older adults. The larger effect size was observed with the shorter (<6 weeks) intervention, while the longer intervention (≥6 weeks) produced a smaller effect. The substantial heterogeneity in the <6 weeks subgroup suggests potential influences from variations in study design, participant characteristics, or assessment tools.

#### 3.5.3 Subgroup analyses of the effects of exercise combined with tDCS on cognitive function in older adults with different cognitive levels

**Figure 6:**
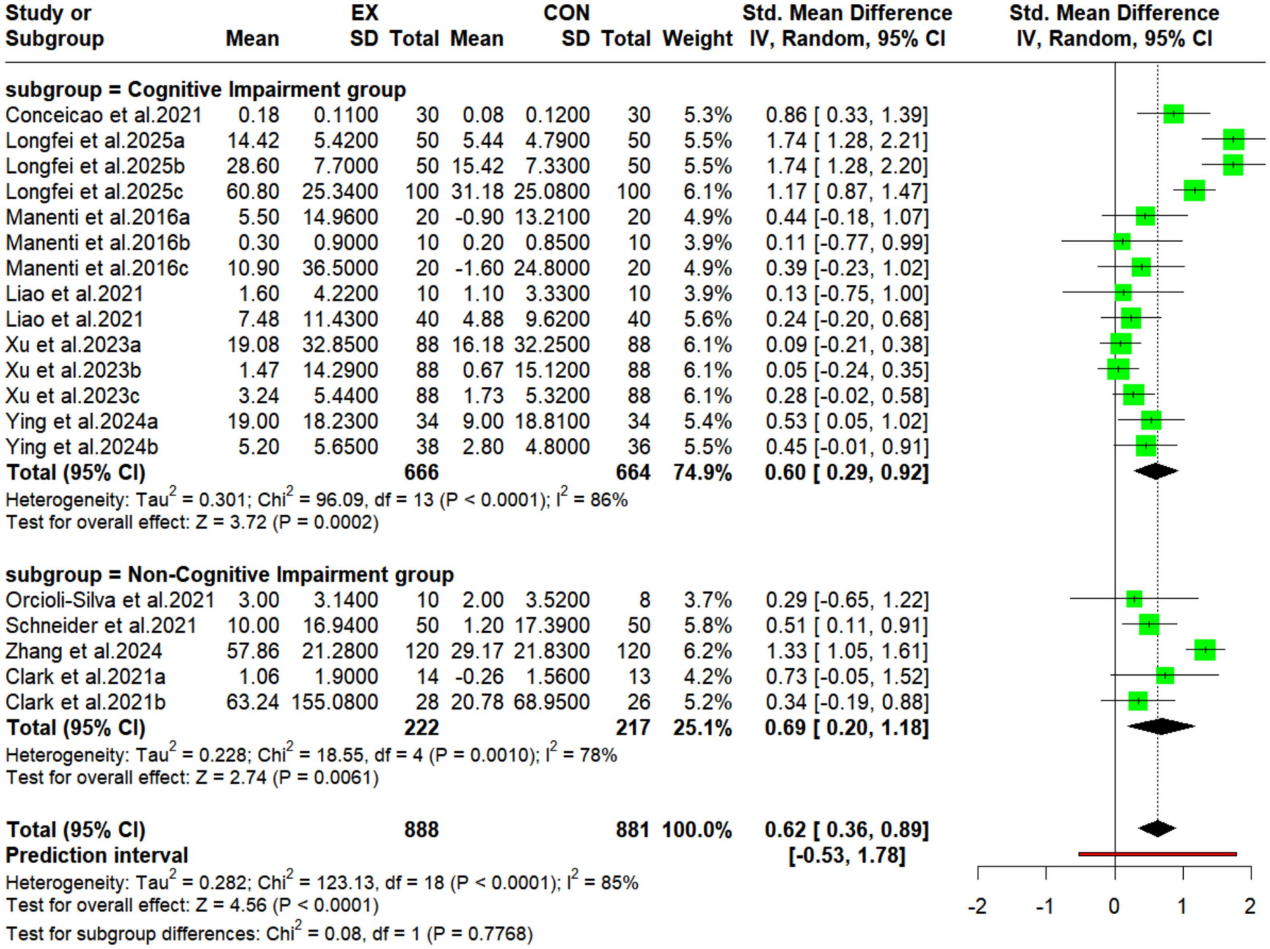
Subgroup analyses forest plot showing the effects of motor-associated tDCS on cognitive function in older adults with different types of cognitive impairment.

**Figure 7:**
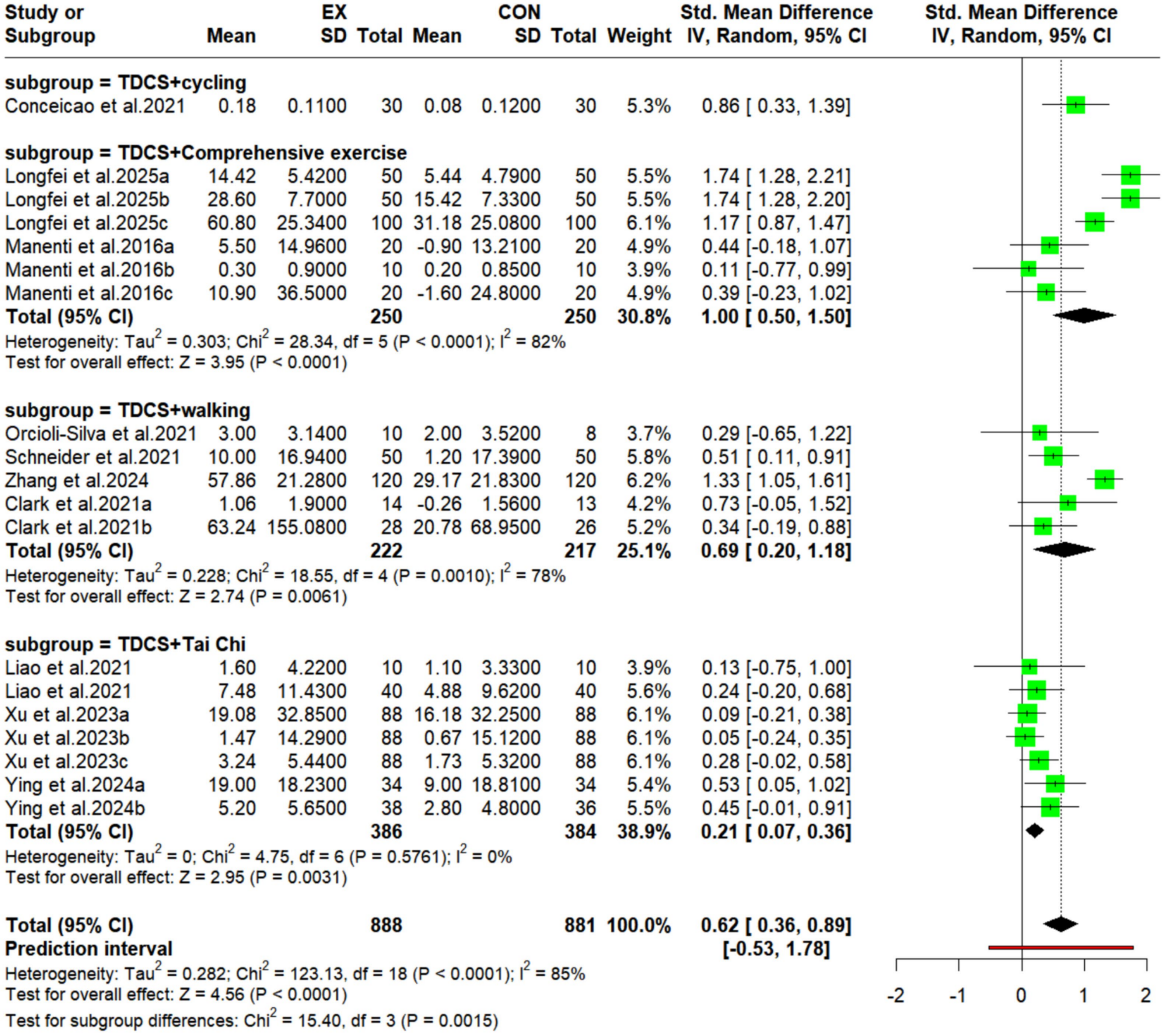
Subgroup analysis of the effects of different types of exercise combined with tDCS on the cognitive function of older adults.

This study analyzed the intervention period by dividing participants into two subgroups: the Cognitive Impairment group and the Non-Cognitive Impairment group (Figure 6). The analysis demonstrated a pooled effect size of SMD = 0.60 (95% CI: 0.29, 0.92; *p* = 0.0002) for the Cognitive Impairment group (experimental: *n* = 666; control: *n* = 664). The Non-Cognitive Impairment group (experimental: *n* = 222; control: *n* = 217) showed a pooled effect size of SMD = 0.69 (95% CI: 0.20, 1.18; *p* = 0.006). High heterogeneity was observed in both subgroups (Cognitive Impairment: I^2^ = 86%; Non-Cognitive Impairment: I^2^ = 78%). These results indicate that exercise combined with tDCS effectively improves cognitive function in the elderly population. The substantial heterogeneity suggests that future studies should prioritize methodological standardization of stimulation protocols and cognitive assessment criteria to enhance evidence reliability (Figure 7).

#### 3.5.4 Subgroup analysis of the effects of different types of exercise combined with tDCS on cognitive function in older adults

Subgroup analysis revealed that different exercise types significantly modulated the intervention effect of tDCS (*p* = 0.0015). Among them, tDCS combined with comprehensive exercise yielded the largest effect size (SMD = 1.74, 95% CI: 1.28–2.21, *p* < 0.0001), albeit with high between-study heterogeneity (I^2^ = 82%). The next largest effects were observed for tDCS combined with cycling (only one study; SMD = 0.86, 95% CI: 0.33–1.39, *p* = 0.0015) and tDCS combined with walking (SMD = 0.64, 95% CI: 0.18–1.10, *p* = 0.0061), the latter showing moderate-to-high heterogeneity (I^2^ = 78%; heterogeneity for cycling not applicable). tDCS combined with Tai Chi Chuan produced the smallest but most robust effect (SMD = 0.25, 95% CI: 0.09–0.42, *p* = 0.0031), with no heterogeneity (I^2^ = 0%).

### 3.6 Regression analysis of the effects of different exercise parameters and tDCS parameters on cognitive function in older adults

**Figure 8:**
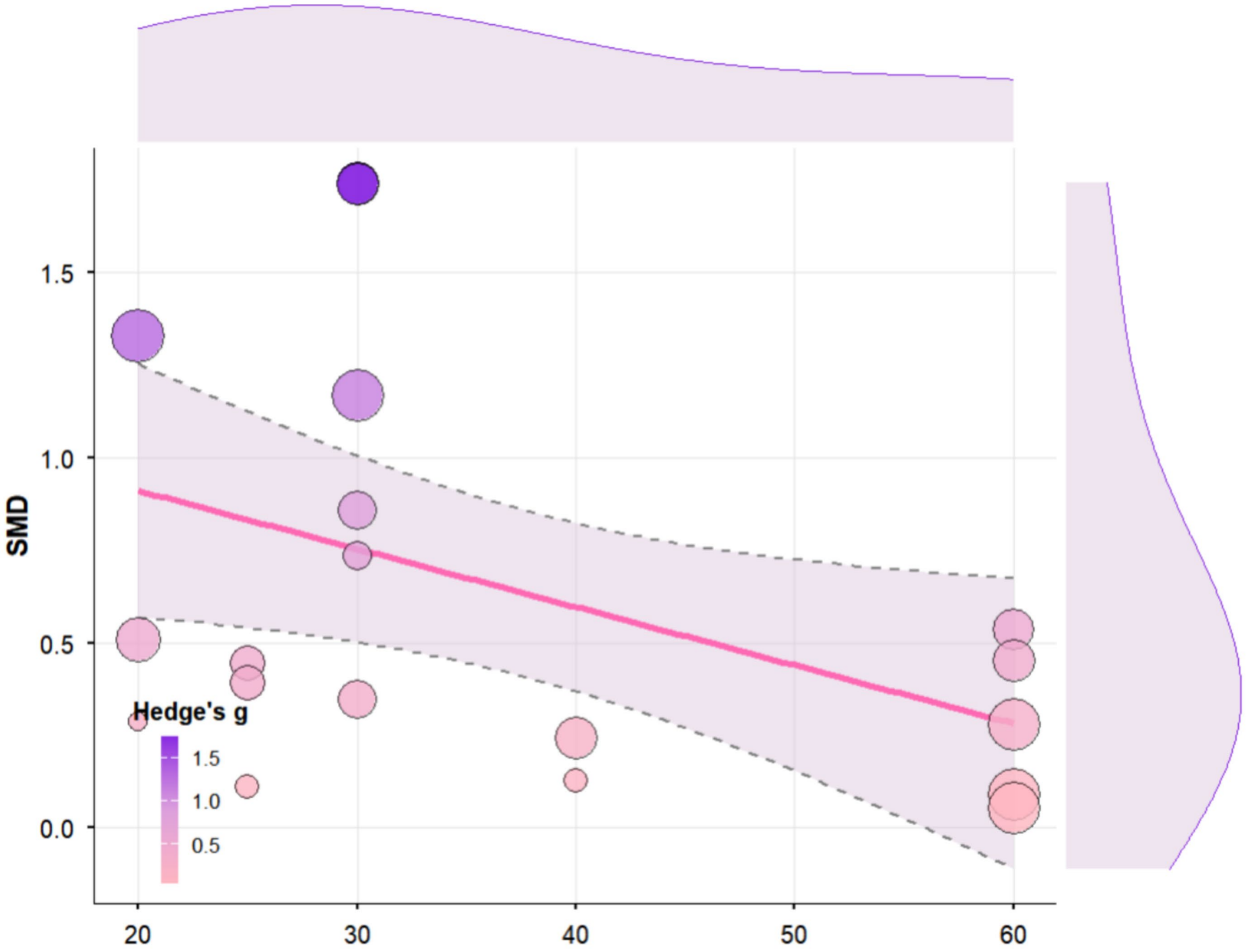
Regression analysis of the effect of tDCS stimulation time on cognitive function in Older Adults.

Regression analysis revealed that tDCS stimulation time had a significant positive moderating effect on cognitive function in elderly people (b = 0.09, SE = 0.03, 95% CI [0.04, 0.14], *p* = 0.0002; Figure 8). Specifically, each unit increase in stimulation time was associated with a 0.0936 increase in the average effect size. According to the regression equation, the predicted effect size shifted from negative to positive when stimulation time reached approximately 15.6 units. Heterogeneity analysis indicated significant residual heterogeneity among studies (QE(17) = 70.90, *p* < 0.0001, I^2^ = 70.8%), with tDCS stimulation time accounting for approximately 50.6% of the inter-study heterogeneity (R^2^ = 50.59%).

**Figure 9:**
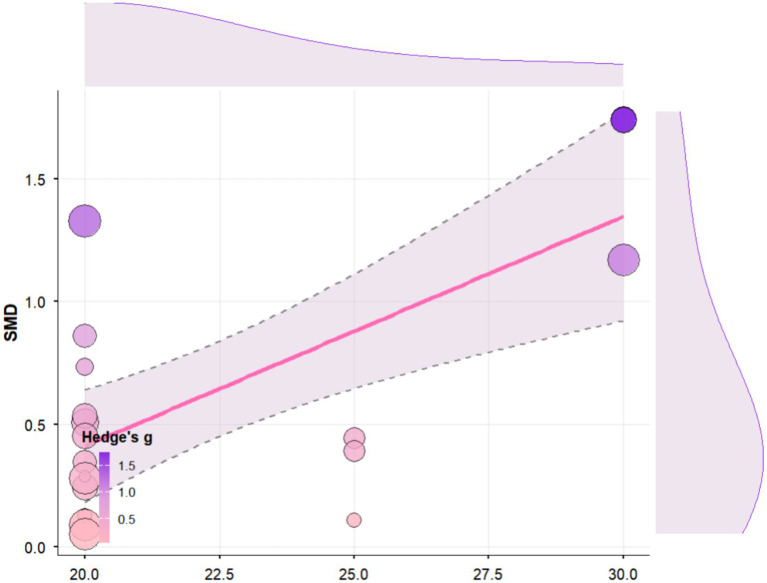
Regression analysis of the impact of single exercise time on cognitive function in older adults.

Regression analysis revealed that the combined intervention period (in weeks) had a significant negative moderating effect on cognitive function in older adults (b = −0.0551, SE = 0.0253, 95% CI [−0.12, −0.01], *p* = 0.0297; Figure 9). Specifically, each additional week of the intervention period was associated with an average decrease of 0.05 in the cognitive effect. Heterogeneity analysis indicated significant residual heterogeneity among the included studies (QE(17) = 71.30, *p* < 0.0001, I^2^ = 78.42%), with the combined intervention period accounting for approximately 26.28% of the inter-study heterogeneity (R^2^ = 26.28%).

**Figure 10:**
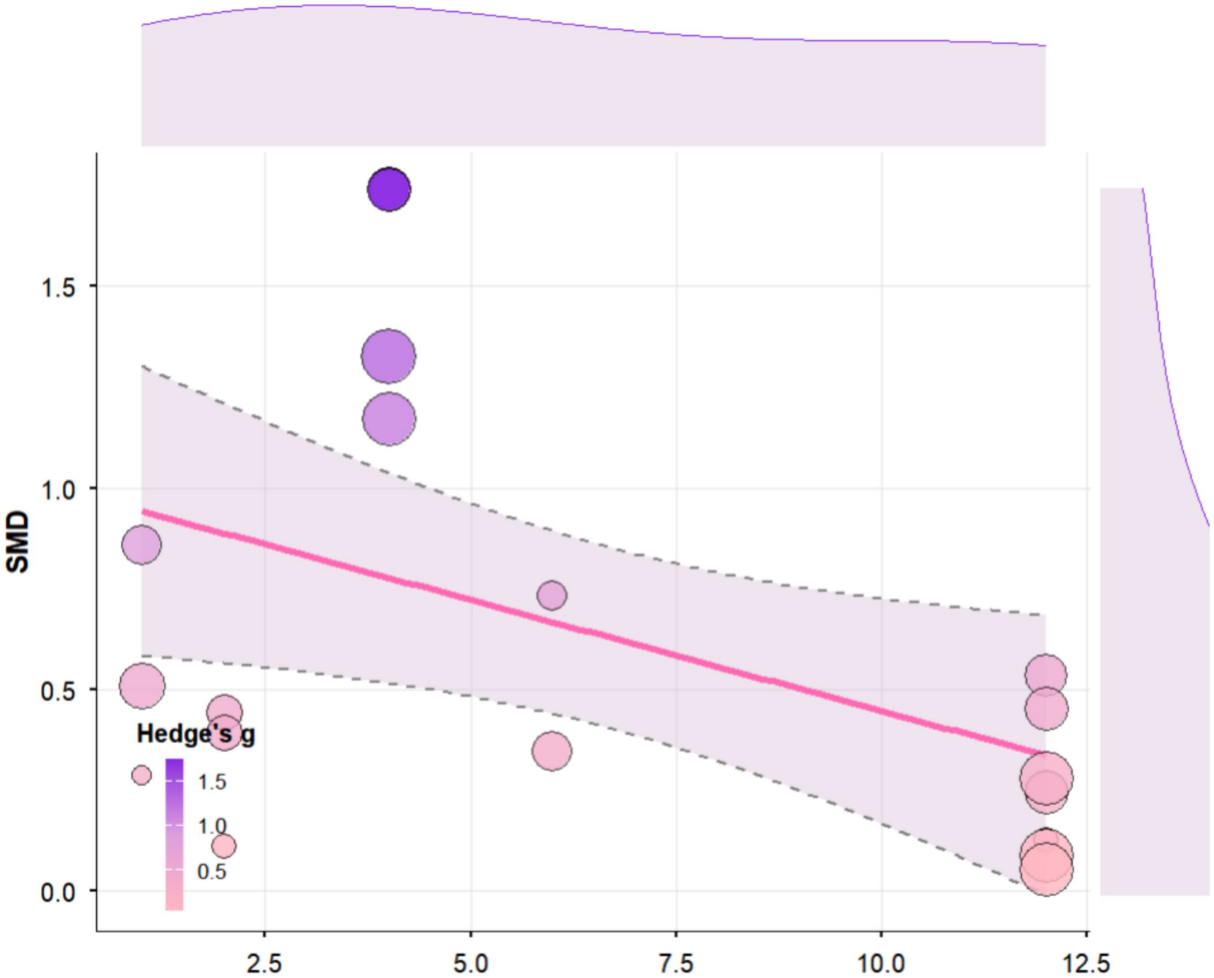
Regression analysis of the impact of combined intervention cycles on cognitive function in elderly people.

Regression analysis revealed that single exercise time had a significant negative moderating effect on cognitive function in elderly individuals (b = −0.02, SE = 0.01, 95% CI [−0.03, −0.001], *p* = 0.0335; Figure 10). Specifically, each one-unit (e.g., minute) increase in single exercise time was associated with an average decrease of 0.0157 in the cognitive effect. Heterogeneity analysis indicated significant residual heterogeneity among studies (QE(17) = 68.18, *p* < 0.0001, I^2^ = 78.25%), with single exercise time explaining approximately 25.78% of the between-study heterogeneity (R^2^ = 25.78%).

### 3.7 Publication bias test

**Figure 11:**
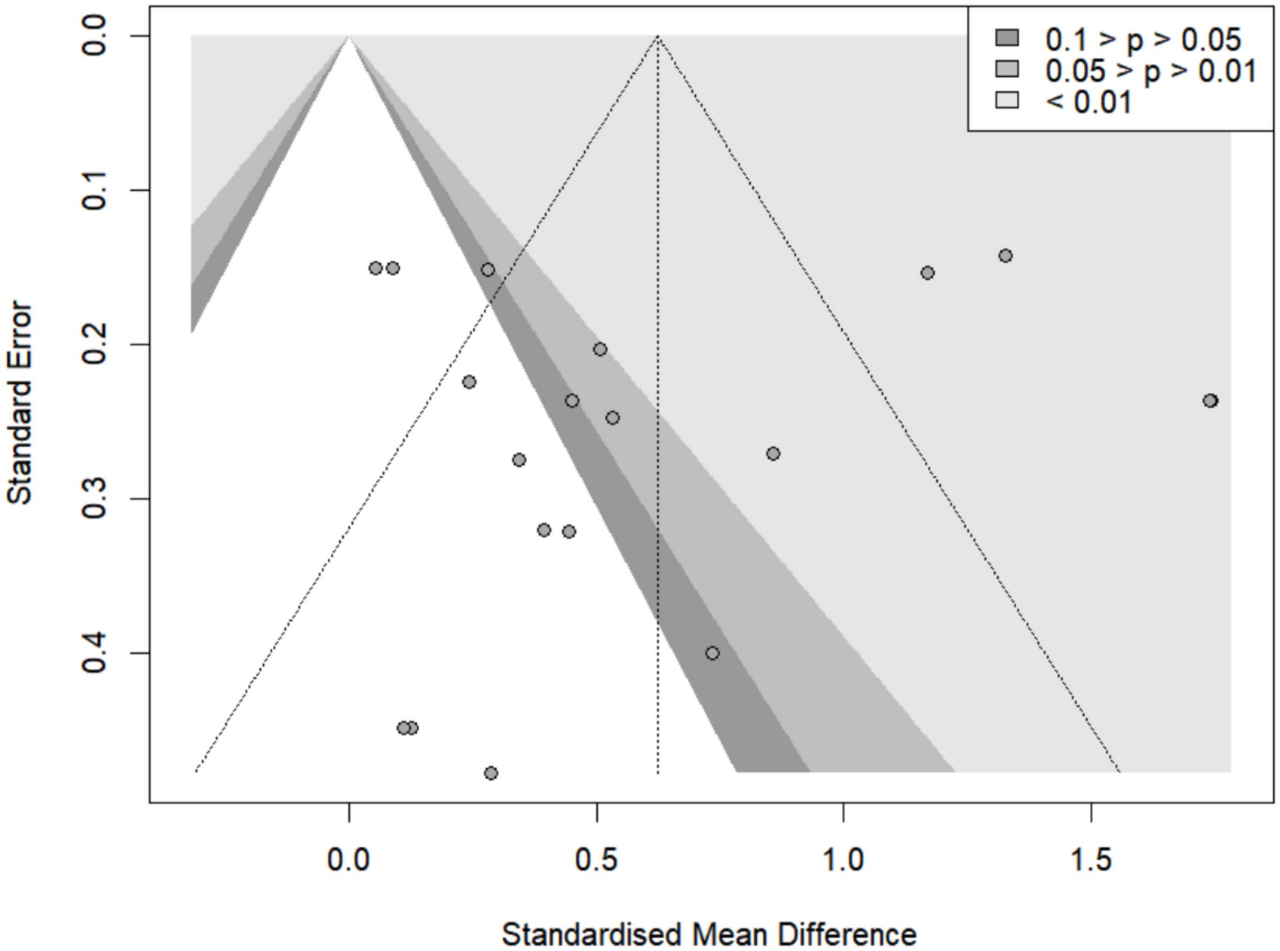
Overall funnel plot of the effects of exercise combined with tDCS on cognitive function in elderly individuals.

As shown in Figure 11, the funnel plot exhibits some asymmetry, suggesting potential publication bias. To evaluate potential publication bias in the meta-analysis, the linear regression test for funnel plot asymmetry (Egger’s test) was performed, with the standard error as the predictor and inverse variance as the weight, accounting for multiplicative residual heterogeneity (τ^2^ = 7.220). The test yielded a biased estimate of −0.41 (SE = 1.7695). The result was not statistically significant (t = −0.23, df = 17, *p* = 0.8188), indicating no evidence of funnel plot asymmetry. Therefore, publication bias is unlikely to have a major impact on the overall findings of this meta-analysis.

### 3.8 Sensitivity analysis

A sensitivity analysis was performed by sequentially excluding each of the 10 included studies and altering the analytical methods to reassess the effect sizes. The results demonstrated that excluding individual studies did not significantly alter the pooled effect estimates, indicating the robustness of this meta-analysis.

### 3.9 GRADE evidence level assessment

**Figure 12:**
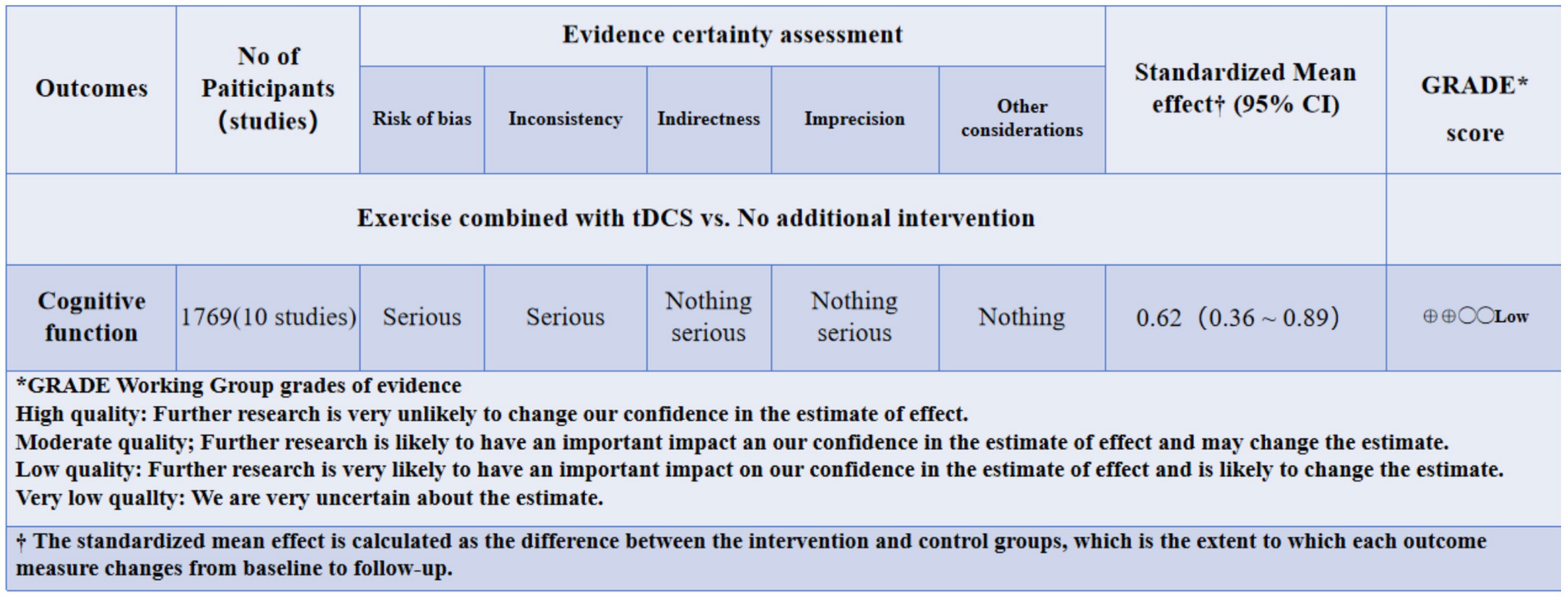
GRADE evidence level assessment.

At the level of evidence evaluation, the effect of exercise combined with tDCS on improving cognitive function in elderly people was rated as “moderate” compared to not exercising (Figure 12).

## 4 Discussion

### 4.1 Mechanistic pathways of exercise combined with tDCS for improving cognitive function in older adults

As humans age, the brain undergoes significant neurophysiological changes. These include reductions in brain volume (particularly within the prefrontal cortex and hippocampus), grey matter atrophy, decreased secretion of neurotransmitters such as acetylcholine and dopamine, and impaired synaptic efficiency (Yang et al., 2023). Functionally, brain activation patterns reorganize, characterized by reduced lateralization (e.g., increased bilateral prefrontal cortex activation during memory retrieval) and compensatory increases in prefrontal cortex activity coupled with decreased activity in posterior brain regions. Concurrently, connectivity within resting-state neural networks, particularly the default mode network, diminishes (Shengmin, 2018). Furthermore, damage to long-range white matter fibres impairs inter-regional brain coordination. Collectively, these structural degenerative changes, along with functional compensatory mechanisms and dysfunctions, constitute the neural basis for cognitive decline in older adults.

Transcranial direct current stimulation, a non-invasive neurostimulation technique, is characterized by its ability to regulate cortical excitability, synaptic plasticity, and functional brain network connectivity. This technique can improve human cognitive functions, including perception, working memory, attention, motor learning, and decision-making (Giordano et al., 2017; Na and Xue, 2024). Additionally, exercise has been demonstrated to positively alter neurotransmitter levels and brain structure in older adults (Yi et al., 2022; Yang et al., 2023). Consequently, the potential synergistic benefits of combining exercise with tDCS for enhancing cognitive function in older adults warrant further exploration.

#### 4.1.1 Possible mechanisms by which tDCS improves cognitive function

Transcranial direct current stimulation delivers low-intensity direct current (typically between 0.5 and 2 mA) to the cerebral cortex via electrodes, creating a current loop between the anode and cathode to modulate brain activity. Typically, anodal stimulation enhances cortical neuron excitability, whereas cathodal stimulation reduces it. The specific mechanisms by which tDCS influences brain activity are complex and multifactorial and have not yet been fully elucidated. The primary hypothesized mechanisms involve the alteration of neuronal resting membrane potentials, modulation of synaptic function, and activation of functional brain network connectivity.

The primary mechanism involves modulating neural membrane potential. Anodal tDCS depolarises the resting membrane potential of neurons, thereby enhancing their excitability; conversely, cathodal stimulation induces hyperpolarisation, reducing neuronal excitability (Brunoni et al., 2012; Nitsche and Paulus, 2001). These subthreshold membrane potential changes (anodal: depolarisation; cathodal: hyperpolarisation) underlie tDCS regulation of cortical excitability (Nitsche and Paulus, 2001). Specifically, anodal tDCS at 1–2 mA can depolarise the resting membrane potential by approximately 0.2–0.5 mV (Opitz et al., 2016). This transient increase in membrane excitability enhances the probability of action potential (AP) generation, thereby increasing the spontaneous neuronal firing rate. In contrast, cathodal tDCS produces transient hyperpolarisation (Romero et al., 2014; Nitsche et al., 2012), consequently reducing cortical excitability and spontaneous neuronal firing. While these mechanisms account for the immediate effects of tDCS, the persistence of stimulation effects for up to an hour requires further elucidation through more precise mechanisms.

The second proposed mechanism involves alterations in synaptic plasticity. tDCS modulates synaptic transmission strength by influencing mediators within the synaptic microenvironment, thereby inducing synaptic modifications. For instance, tDCS enhances N-methyl-D-aspartate (NMDA) receptor activity and alters GABAergic function (Brunoni et al., 2012; Park et al., 2014). Gamma-aminobutyric acid (GABA), a key mediator of cortical synaptic plasticity, exhibits accelerated release rates during cortical stimulation. This reduces cortical excitability and facilitates synaptic remodeling (Stagg et al., 2009a). Monai et al. (2016) further demonstrated that tDCS-induced synaptic plasticity alterations correlate with changes in astrocytic calcium ion concentrations. Regarding neuromodulatory effects, anodal tDCS decreases GABA while increasing glutamate concentrations, whereas cathodal stimulation produces the opposite effect (Stagg et al., 2009b). tDCS promotes synaptic plasticity by regulating the synaptic microenvironment and modifying systems, including dopaminergic pathways (Liebetanz et al., 2002; Nitsche et al., 2003). This mechanism parallels long-term potentiation and depression in learning, explaining tDCS’s prolonged effects. Neurotransmitters contribute significantly to tDCS outcomes, potentially improving working memory and executive function to alleviate cognitive impairment (Hama et al., 2017). Furthermore, sustained tDCS benefits may involve neuroplasticity processes mediated by dopamine (Monte-Silva et al., 2009), serotonin (Nitsche et al., 2009), acetylcholine (Kuo et al., 2007), BDNF (van der Vliet et al., 2018), and other neurotransmitters.

The third mechanism involves the modulation of the brain’s functional network connectivity. tDCS influences cortical and subcortical activity through long-distance functional connectivity (Na and Xue, 2024). Specifically, anodal tDCS enhances functional coupling between stimulated regions and connected brain areas, improving associated cognitive functions and behavioral control. Resting-state network studies demonstrate tDCS modulation of local brain activity alongside interhemispheric and intrahemispheric functional connectivity (Kunze et al., 2016; Bouchard et al., 2023). For instance, high-definition anodal tDCS (1.5 mA, 20 min) targeting the right inferior frontal gyrus significantly increases its resting-state functional connectivity with the left prefrontal cortex (Muller et al., 2023). The default mode network (DMN) and frontoparietal network (FPN)—key networks regulating attention and memory—show enhanced functional connectivity between DMN and bilateral FPN following anodal tDCS (2 mA, 20 min) over the left dorsolateral prefrontal cortex (DLPFC) (Keeser et al., 2011). This suggests DLPFC stimulation, as a hub node in both networks, modulates coherence across functional networks during rest. Additionally, fMRI studies reveal that left or right DLPFC anodal tDCS (2 mA, 20 min) enhances task-positive network synchrony while reducing DMN synchrony (Pena-Gomez et al., 2012). This DMN reduction may optimize cognitive resource allocation by enhancing local neural efficiency. Widespread functional network reorganization following a single anodal tDCS session has also been observed (Ardolino et al., 2005). Neuroimaging evidence includes fNIRS-detected prefrontal hemodynamic changes (Merzagora et al., 2010) and EEG-documented tDCS modulation of brain oscillatory activity, particularly frequency-specific oscillations (Jacobson et al., 2012).

#### 4.1.2 Possible mechanisms by which exercise improves cognitive function

Exercise is a simple yet effective strategy for maintaining brain function, serving as an indispensable component of daily life, crucial for promoting brain health and enhancing quality of life. Exercise improves cognitive function and reduces the risk of neurological disorders by promoting neurogenesis and regeneration, enhancing synaptic plasticity, regulating neuronal activity, and restoring homeostasis of small molecules within the neuronal extracellular environment. Consequently, exercise constitutes a vital non-pharmacological therapy for protecting brain health and preventing neurodegenerative diseases (Hortobágyi et al., 2022; Mellow et al., 2020; Ruegsegger and Booth, 2018; la De Rosa et al., 2020; Chen et al., 2022). Furthermore, exercise effectively enhances neural plasticity—the nervous system’s capacity to adapt to environmental changes. Understanding this multi-level regulation across scales, from molecular and cellular to circuit and system levels, is fundamental for elucidating exercise-mediated neuroprotective mechanisms and cognitive intervention strategies.

Regular exercise induces beneficial changes in neuronal activity within the brain. It regulates neuronal firing patterns and frequencies, enhancing information encoding efficiency in the motor cortex while optimizing the excitation-inhibition balance, thereby improving the overall functional state of neural networks (Marino et al., 2023). Specifically, exercise modulates neural function by altering neuronal firing rates. Research demonstrates that regular exercise increases the spontaneous firing frequency of layer 5 pyramidal neurons in the motor cortex, intensifies their response to motor input, and accelerates their reaction speed during movement initiation (Destexhe and Marder, 2004). Exercise intrinsically heightens the excitability of motor cortex neurons. Furthermore, complex motor tasks prove more effective than simple repetitive movements at inducing prefrontal cortex firing pattern restructuring, bolstering its role in decision-making, attention, and executive control (Chen et al., 2019). Regular exercise also reshapes neuronal firing patterns. For instance, it elevates the proportion of burst firing in cortical neurons, facilitating rapid information transmission and synaptic plasticity. Following aerobic exercise, the proportion of pyramidal cells exhibiting burst firing in the motor cortex significantly increases, concurrent with disinhibition (reduced firing frequency of inhibitory neurons). This shift indicates a movement of the excitation-inhibition balance towards excitation, promoting faster motor information processing (Thacker et al., 2019). Exercise further protects brain function by accelerating the clearance of metabolites from the extracellular space (ECS). Studies reveal that 5 weeks of voluntary wheel running accelerates glymphatic system flow in the mouse brain, enhancing metabolite clearance efficiency and increasing aquaporin 4 (AQP4) expression in astrocytes. These changes help reduce *β*-amyloid (Aβ) deposition and neuroinflammation, maintain synaptic function, and mitigate declines in spatial cognition (Li et al., 2024). Exercise also modulates extracellular neurotransmitter concentrations to confer neuroprotection. For example, it ameliorates motor dysfunction in Parkinson’s disease (PD) model rats by reducing striatal extracellular glutamate (Glu) concentrations (Shi et al., 2019). Additionally, exercise elevates 5-hydroxytryptamine (5-HT) levels in the brain and raphe nucleus while enhancing the expression of the serotonin transporter (SERT) and 5-HT receptors in the cortex and hippocampus (Pietrelli et al., 2018). Exercise training can enhance cortical-striatal plasticity and increase dopamine release (Feng et al., 2020). Age-related cognitive decline associated with hippocampal atrophy correlates with reduced brain-derived neurotrophic factor (BDNF) levels; improving BDNF levels can partially counteract this decline (Buchman et al., 2016). A meta-analysis by Zhou et al. (2022) further confirms that diverse exercise modalities—including aerobic training, high-intensity interval training, multi-modal training, resistance training, and combinations thereof—significantly elevate serum BDNF levels across various populations.

Exercise can effectively enhance the plasticity of neural synapses in the brain. Synaptic structural plasticity refers to the ability of synaptic connections between neurons to undergo dynamic changes in both form and function. Abnormalities in synaptic plasticity are an important component of the pathological characteristics of neurodegenerative diseases such as Alzheimer’s disease (AD) and Parkinson’s disease (PD). Exercise can alter the size, number, and formation of new synaptic connections, as well as modify the probability of presynaptic neurotransmitter release and the postsynaptic sensitivity to neurotransmitter release, thereby inducing changes in synaptic transmission efficiency. This promotes the generation, strengthening, and maintenance of synapses in brain regions such as the cerebral cortex, hippocampus, and striatum, thereby improving learning, motor, and cognitive functions (la De Rosa et al., 2020; Dejanovic et al., 2024). Exercise enhances synaptic transmission efficiency by regulating key synaptic proteins. It increases cortical PSD-95 and SNAP-25 expression, promoting motor skill learning; simultaneously, through SNAP-91 lactylation modification, it enhances presynaptic vesicle numbers, synaptic protein levels, and PSD length in the medial prefrontal cortex (mPFC), alleviating anxiety-related behaviour. However, exercise fatigue reduces striatal PSD thickness (Chen et al., 2022). Exercise can increase dendritic spine density by regulating the turnover process of dendritic spines (including their generation, elimination, and rate). In Parkinson’s disease (PD) models, exercise increases the density of dendritic spines in medium spiny neurons (MSNs) in the striatum, thereby improving motor dysfunction (Palasz et al., 2019). Exercise can also regulate synaptic transmission efficiency. In Alzheimer’s disease (AD) models, exercise improves cognitive impairment by increasing the amplitude and frequency of mEPSCs and mIPSCs in prefrontal cortical pyramidal neurons (Mu et al., 2022).

Exercise regulates neural network changes in the brain. It delays age-related cognitive decline by enhancing the integrity of the right-sided sensorimotor network (Boa et al., 2024). Furthermore, exercise improves global cognitive function, attention, executive function, and language ability in older adults. These benefits are associated with increased functional connectivity within several key regions: the dorsolateral prefrontal cortex, the left medial prefrontal cortex, the right anterior cingulate cortex, the default mode network, the left inferior parietal lobe, the left inferior temporal lobe, the inferior frontal gyrus, and the medial executive control network (Boa et al., 2024). Exercise also modulates activation levels in brain regions linked to higher cognitive functions (Prakash et al., 2015). Specifically, exercise-induced reductions in prefrontal cortex activation indices, including decreased oxygenated haemoglobin and total haemoglobin in the left prefrontal cortex, correlate with improved cognitive task performance (Coetsee and Terblanche, 2017). Research indicates that the prefrontal cortex (particularly the orbitofrontal and dorsolateral regions) shows elevated activation during both slow and fast movements, influencing inhibitory control and working memory (Guerin et al., 2023). Increased activation in the striatum and insula is beneficial for movement-related learning and attention control (Ji et al., 2017). Additionally, exercise influences brain network activation levels (Rajab et al., 2014). Physical exercise alters the activation patterns of the default mode network (DMN) and the fronto-executive network (FEN), leading to enhanced central executive function in older adults (Voss et al., 2010). Exercise can also improve the synchrony of activity within auditory-related brain networks (Rajab et al., 2014).

#### 4.1.3 Synergistic mechanism of exercise combined with tDCS on cognitive function

Combined motor intervention and tDCS can produce synergistic enhancement effects, underscoring the critical influence of brain state during stimulation. Multiple studies demonstrate that both tDCS and motor interventions improve cognitive function by modulating cortical excitability and neural plasticity (Steinberg et al., 2019; Talar et al., 2022; Xu et al., 2023; Zhang et al., 2024; Nguyen et al., 2024). Given that exercise and tDCS share common neural substrates, it is hypothesised that combining these techniques produces synergistic effects, yielding cognitive enhancement outcomes superior to those of single interventions. This combined strategy is well-established. Recent studies confirm that tDCS and exercise can produce complementary effects under combined intervention conditions (Steinberg et al., 2019; Talar et al., 2022; Nguyen et al., 2024; Marchiotto et al., 2025). Similarly, other combined intervention models, such as tDCS integrated with cognitive therapy, demonstrate additive effects. This modality uses tDCS to modulate cortical excitability and promote neuroplasticity alongside cognitive training to enhance brain function and facilitate recovery (Uehara et al., 2025). Thus, tDCS may synergistically regulate neural network combinations to improve cognitive function and alter neural activity. Research indicates that anodal tDCS (A-tDCS) applied to the primary motor cortex (M1) enhances its structural and functional plasticity, augmenting the effects of physical exercise (Marchiotto et al., 2025). Combining tDCS with physical activity also enhances *θ*-frequency synchrony within the motor cortex, a hallmark of motor network connectivity (Marchiotto et al., 2025; Noga et al., 2017). Notably, although tDCS and exercise act on overlapping brain regions, they operate through distinct molecular mechanisms. tDCS may improve cognition by altering acetylcholine, dopamine, and GABA levels, as well as cortical activation. Conversely, exercise modulates levels of growth factors (IGF-1, BDNF, and VEGF), dopamine, glutamate, serotonin, and norepinephrine, while also promoting vascularisation and neurogenesis (Yang et al., 2023; Steinberg et al., 2019).

The combination of motor tasks and tDCS interventions for cognitive function can be categorized into two types: direct (e.g., synchronous cognitive training during motor activity) and indirect (e.g., sequential administration). Further insights into determining optimal intervention timing derive from the study by Parkin et al. (2015). This study proposed priming or a ‘preconditioning stimulus’—a brief stimulus applied before the main task or tDCS intervention. tDCS can enhance the effects of subsequent interventions by inducing metaplasticity (Parkin et al., 2015). Emerging evidence from motor, cognitive, and visual sciences suggests that metaplasticity not only produces more reliable outcomes but may also enhance the effects of traditional protocols (Hurley and Machado, 2017). The concept of metaplasticity originates from Bienenstock, Cooper, and Munro’s (BCM) synaptic plasticity model (Bienenstock et al., 1982). The BCM theory posits that prior cortical activity modulates the threshold for subsequent responses: inhibitory activity lowers the threshold for subsequent excitatory responses, whereas excitatory activity raises it (Cooper and Bear, 2012). Consequently, neuronal plasticity depends on prior activity history (Hulme et al., 2013). This experience-dependent neural plasticity mechanism is thought to require a rapid, state-dependent system capable of dynamically adapting to prior synaptic activity to maintain network stability and promote persistent changes such as long-term potentiation (LTP) or long-term depression (LTD) (Abraham, 2008). Research demonstrates significant benefits of combining anodal tDCS with motor tasks, as tDCS modulates widespread neuronal activity. This modulation can lead to dopamine release via the basal ganglia-cortical-cerebellar system and other motor networks within motor cortical regions (Nguyen et al., 2024). Furthermore, repeated anodal tDCS sessions can directly induce plasticity-related long-term potentiation in the human cortex.

The pre-interventional state of a neural network can influence the efficacy of subsequent interventions targeting that network. Carvalho et al. (2015) investigated this mechanism using a working memory task (N-back), applying two tDCS sessions with opposite polarities to the dorsolateral prefrontal cortex (DLPFC) separated by a time interval (Carvalho et al., 2015). Their findings demonstrated that anodal tDCS applied as a pre-treatment weakened the subsequent facilitatory effect of anodal tDCS (the conditioned stimulus) on working memory. Crucially, this attenuation occurred only when a delay separated the two sessions, suggesting that the pre-treatment reversed the polarity effect direction of the subsequent stimulus. Conversely, applying anodal tDCS as a pre-treatment immediately before (i.e., 10 min before) the conditioned anodal tDCS enhanced its effect on working memory. This pattern indicates that the initial inhibitory period induced by anodal tDCS may trigger compensatory upregulation via metaplasticity mechanisms, thereby reversing its subsequent effect. These results align with the Bienenstock-Cooper-Munro (BCM) model for maintaining network stability and the ‘rebound effect’ phenomenon (Rahman et al., 2013). Furthermore, acute exercise interventions (e.g., short-term high-intensity exercise) rapidly enhance brain excitability and brain-derived neurotrophic factor (BDNF) levels, serving as effective pre-treatments for subsequent anodal or cathodal tDCS. Exercise may promote long-term potentiation (LTP)-like plasticity by synergizing with neurogenesis mechanisms. Thus, both exercise and tDCS can be viewed as tools for inducing specific optimal brain states (neural priming): exercise acts as a global activator regulating overall brain excitability, while tDCS enables precise targeting of specific brain regions for therapeutic intervention. Additionally, engaging in exercise within hours after tDCS may enhance the consolidation of intervention effects.

The inhibitory system represents another potential mechanism underlying the combined effects of motor tasks and tDCS on cognitive function. Recent evidence suggests that enhancing inhibitory tone is crucial for improving cognitive abilities due to its key regulatory role across multiple competing neural systems; conversely, insufficient cognitive demands may weaken inhibitory function, potentially related to compensatory mechanism failure (Capano et al., 2015). Additionally, prefrontal cortex tDCS may enhance neural circuit efficiency, thereby improving cognitive performance and reducing the attentional resources required for specific cognitive tasks. This resource reduction frees attentional resources for concurrent tasks (Liao et al., 2021). According to the dual-task bottleneck theory, tasks sharing neural resources can cause processing delays (Ruthruff et al., 2001). tDCS-related improvements may therefore stem from enhanced processing speed and reduced inter-task latency. Interestingly, motor intervention exhibits a dual-phase regulatory effect: an initial enhancement of excitatory circuit activity followed by increased inhibitory circuit activity. This characteristic has been utilized to enhance inhibitory control in children, particularly those with attention-deficit/hyperactivity disorder (ADHD) (Chang et al., 2014). Anodal tDCS appears to induce similar effects, causing a transient increase in neuronal spontaneous discharge followed by enhanced cortical inhibition. Furthermore, combining these two interventions may produce synergistic effects. However, since motor intervention efficacy depends on its later inhibitory phase rather than the initial excitatory phase, precise temporal coordination of both interventions is critical.

### 4.2 Effects of exercise combined with tDCS on cognitive function in older adults

This study presents the first systematic meta-analysis assessing the effects of combined exercise and tDCS intervention on cognitive function in elderly populations. Based on the 10 included studies, this combined intervention improves global cognitive function in the elderly. However, its effects vary across specific cognitive dimensions, including executive function and memory. Furthermore, the extent of cognitive improvement is influenced by both the intervention duration and the participants’ baseline cognitive level, aligning with previous research findings (Steinberg et al., 2019; Talar et al., 2022; Marchiotto et al., 2025). Cognitive enhancement involves augmenting one or more core brain abilities. Research indicates tDCS has broad applications in enhancing basic and advanced cognitive functions. For example, Boggio et al. (2009) demonstrated that anodal tDCS stimulation of the left dorsolateral prefrontal cortex (DLPFC) and temporal cortex improved visual recognition memory in Alzheimer’s disease (AD) patients. Additionally, tDCS can persistently alter the excitability of cortical neurons in regions like the primary motor cortex, DLPFC, posterior parietal cortex, and inferior frontal cortex, along with their connected networks, representing potential mechanisms for cognitive improvement. Single-session tDCS effectively stimulates the temporal lobe and enhances cortical networks involved in working memory tasks, improving memory in patients with mild cognitive impairment (MCI) and AD, though significant variability exists in electrode placement and treatment response (Liu et al., 2020). Conversely, exercise promotes neurogenesis, enhances synaptic plasticity, improves cognitive function, and reduces neurological disease incidence, making it a key intervention for treating central nervous system disorders and mitigating aging effects (Chow et al., 2022). Coupling tDCS with physical activity can enhance motor cortex activation and functional interactions. Combining tDCS stimulation with challenging walking conditions immediately boosts cortical excitability post-intervention, reducing motor-cognitive interference, as evidenced by decreased dual-task cost (Schneider et al., 2021). Specifically, increased walking speed and complexity progressively activate frontal lobe neural circuits responsible for executive function (Clark et al., 2014), while impaired executive function correlates with slowed gait and reduced complex gait task performance (Steinberg et al., 2019). For patients with specific cognitive deficits, tailored intervention modes remain effective. Studies indicate that Tai Chi combined with tDCS enhances dual-task gait performance and cognitive function in MCI patients (Xu et al., 2023; Liao et al., 2021). Similarly, combining exercise with tDCS positively affects cognitive function post-stroke, yielding synergistic improvements in memory and executive function (Longfei et al., 2025). In Parkinson’s disease (PD) patients, applying anodal tDCS to the prefrontal cortex (PFC) during walking immediately improves gait variability, processing speed, and executive control (Conceição et al., 2021). Therefore, the combined exercise and tDCS intervention positively affects cognitive function in the elderly. Building on the potential synergistic effects of exercise and tDCS, we explore how this combined model can enhance the impact of personalized treatment on cognitive function and provide disease-specific strategies to mitigate age-related cognitive decline progression.

### 4.3 Effects of combined exercise combined with tDCS on cognitive function in older adults with different cognitive levels

Based on a subgroup analysis of distinct cognitive dimensions in the elderly, this study categorized participants into three subgroups: executive function, memory, and global cognitive function for analysis. The results indicate that the combined intervention model has the greatest impact on the overall cognitive function of the elderly, followed by executive function. The impact on memory function is not significant. Consequently, conclusions derived from the literature review warrant further investigation. It is hypothesized that the comprehensive effects of interventions are more readily detectable in global cognitive assessments integrating multiple dimensions. Given that the prefrontal cortex, which regulates executive function, is more vulnerable to aging, enhancing its neuroplasticity likely requires higher-intensity, targeted training. Global cognitive scales demonstrate high sensitivity to the cumulative effects of multi-dimensional, subtle improvements, whereas executive function-specific tests possess relatively lower sensitivity for detecting complex, higher-order cognitive changes (i.e., higher detection thresholds). Research indicates that combined motor and tDCS intervention is effective for improving executive function, memory function, and global cognitive function (Steinberg et al., 2019; Talar et al., 2022). Given the significant role of the prefrontal cortex (PFC) in cognitive processing and executive function, numerous studies demonstrate that tDCS-induced modulation of PFC activity enhances cognitive performance (Steinberg et al., 2019). Research further indicates that single-session tDCS stimulation targeting specific brain regions (e.g., prefrontal cortex, sensory cortex, parietal cortex) can effectively modulate cognitive functions like perception, learning, and memory in healthy individuals (Da-long et al., 2018). Anodal tDCS stimulation of the DLPFC increases local cerebral blood flow perfusion, enhances synaptic neurotransmitter release and transmission, strengthens functional connectivity within cortical neural networks, and promotes executive function recovery (Longfei et al., 2025). Additionally, the combination of Tai Chi and tDCS benefits global cognitive function and memory in MCI patients, increases ERP-P300 amplitude, and correlates changes in global cognitive function with P300 (Fz region) amplitude changes (Ying et al., 2024).tDCS may potentiate the positive effects of Tai Chi on gait, potentially extending to cognitive function in MCI patients (Liao et al., 2021). Furthermore, improvements in global cognitive function and memory performance in Parkinson’s disease patients occurred only after combined intervention and remained stable during follow-up (Manenti et al., 2016). In summary, the combined intervention model represents a novel therapeutic strategy for enhancing cognitive function in the elderly.

### 4.4 Effects of different intervention cycles of exercise combined with tDCS on cognitive function in older adults

Based on subgroup analyses categorizing intervention periods into “<6 weeks” and “≥6 weeks,” this study found that exercise combined with tDCS effectively improves cognitive function in older adults. The 6-week intervention demonstrated significantly greater efficacy compared to longer durations (≥6 weeks), which showed diminished effects. High heterogeneity was observed between these subgroups, suggesting potential influences from variations in study design, population characteristics, or assessment tools. While research indicates cognitive benefits in older adults vary with exercise duration (Dhahbi et al., 2025), the dose–response relationship for combined exercise-tDCS interventions remains unclear. Chinese clinical guidelines recommend a standard 3-week tDCS treatment course, adjustable based on individual patient needs (Programme CEGO et al., 2025). Extended interventions may reduce compliance due to exercise fatigue or cumulative tDCS scalp discomfort, or may diminish efficacy through reduced neural adaptability. Cognitive improvements may exhibit a rapid initial response followed by a plateau, with neuroplasticity activation effects potentially saturating after 6 weeks. Significant differences existed among protocols within the ≥6-week subgroup (e.g., 6 vs. 12 weeks), and heterogeneity was further amplified by variations across studies in exercise type, cognitive assessment tools (e.g., MoCA vs. MMSE), and baseline population characteristics (e.g., proportion with MCI). Therefore, the combined intervention may exhibit a ‘time window effect,’ with 6 weeks potentially representing an optimal threshold. Future studies should employ standardized protocols (e.g., uniform tDCS targets, progressive exercise prescriptions) and refined subgroup designs.

### 4.5 Effects of exercise combined with tDCS on cognitive function in elderly individuals with different types of cognitive impairment

Subgroup analysis stratified by cognitive status categorized participants into a ‘cognitive impairment group’ and a ‘non-cognitive impairment group’. The results demonstrated that motor task-concurrent tDCS effectively enhanced cognitive function in the elderly population regardless of baseline cognitive status. However, the magnitude of improvement was significantly greater in the non-cognitive impairment group compared to the cognitive impairment group. Substantial heterogeneity observed between these subgroups underscores the need for future studies to standardize stimulation protocols and cognitive assessment criteria to improve evidence reliability. These findings support the view that motor-concurrent tDCS efficacy varies with cognitive impairment severity, consistent with prior research (Steinberg et al., 2019; Talar et al., 2022). Although the combined intervention improved cognitive function overall, the benefits were more pronounced in the non-cognitive impairment group. Research indicates that individuals with cognitive impairment exhibit impaired neural reserve, reduced neural plasticity, dysfunction in key neurotransmitter systems (e.g., the cholinergic system), and exhausted compensatory mechanisms. These factors significantly constrain responsiveness to neurostimulation interventions like tDCS (Talar et al., 2022). Furthermore, standardized tDCS parameters may inadequately accommodate the altered electric field distribution resulting from structural brain changes (e.g., cortical atrophy) in this population. Additionally, cognitive impairment itself may diminish the depth and quality of engagement during motor tasks, potentially undermining the synergistic effects of the combined intervention (Steinberg et al., 2019). The high subgroup heterogeneity primarily reflects considerable diversity within the ‘cognitive impairment group’ regarding aetiology (e.g., AD, PD, and MCI), disease severity, and comorbidities. Variability within the ‘non-cognitive impairment group’ in baseline cognitive status, coupled with inconsistencies across studies in stimulation protocols, motor interventions, and cognitive assessment tools, collectively contributes to increased effect estimate variability. Studies also indicate that individuals with MCI face a higher dementia risk than cognitively intact individuals (Yi et al., 2022). The baseline cognitive level in older adults influences the efficacy of exercise combined with tDCS for cognitive improvement. In other words, greater cognitive decline presents a greater challenge for reversal and enhancement. Therefore, timely prevention of cognitive decline constitutes the primary and most critical line of defense in the elderly population.

### 4.6 Subgroup analysis of the effects of different types of exercise combined with tDCS on cognitive function in older adults

Our subgroup analysis revealed significant differences in cognitive function improvements among elderly individuals when different types of exercise were combined with tDCS. The combined effect of cognitive-motor dual-task training and tDCS was the largest, albeit with high heterogeneity; conversely, the effect of Tai Chi combined with tDCS was the smallest, yet exhibited zero heterogeneity. According to the systematic review and meta-analysis by Talar et al. (2022) aerobic exercise produces moderate to large improvements in overall cognitive function in healthy elderly individuals and patients with mild cognitive impairment (MCI), but is ineffective in dementia patients. In contrast, tDCS significantly improves cognitive function in dementia patients, suggesting that as cognitive impairment severity increases, the efficacy of peripheral-to-central pathway interventions (e.g., aerobic exercise alone) decreases, whereas direct modulation of cortical excitability (tDCS) becomes more advantageous. Comprehensive exercise achieves maximal effects by combining moderate-to-high intensity aerobic loads with explicit cognitive tasks, thereby activating neurotrophic factor pathways (e.g., the BDNF pathway) and the prefrontal executive function network (Longfei et al., 2025; Zhang et al., 2024). However, the high heterogeneity is attributable to individual differences in physical fitness, cognitive baseline, and dual-task regimens. Tai Chi, in contrast, combines moderate-to-low aerobic intensity with embedded cognitive training (e.g., movement sequences and spatial attention) (Ying et al., 2024; Liao et al., 2021). Although its effect size is small, Tai Chi movements are highly standardized, can be easily implemented simultaneously with tDCS, and stably increase P300 wave amplitude, thus yielding highly consistent effects. In addition, Clark et al. (2021) found that the effect of walking combined with tDCS is modulated by exercise dose. Schneider et al. (2021) confirmed that multi-target tDCS combined with complex walking reduces the dual-task gait cost. Conceição et al. (2021) and Orcioli-Silva et al. (2021) provided a mechanistic explanation from the perspectives of neural efficiency and steady-state plasticity. The overlap and complementarity of aerobic exercise and tDCS in pathways such as BDNF, dopamine, and glutamate provide a biological basis for their synergistic enhancement (Steinberg et al., 2019; Moreau et al., 2015).

In summary, the combined effects of exercise type and tDCS arise from interactions among exercise intensity, cognitive engagement, and disease severity. Comprehensive exercise suits early-stage patients with higher cognitive reserve and physical capacity, whereas Tai Chi combined with tDCS offers unique clinical value for early cognitive decline (e.g., MCI) due to its stability and safety. These findings are exploratory and not evidence of superiority. Although comprehensive exercise showed the largest effect size, high heterogeneity and few studies limit its reliability. Cycling is based on a single study, limiting generalizability. Tai Chi, despite a modest effect size, yielded the most consistent results. Thus, caution is needed, and future high-quality studies are required to validate different exercise-tDCS combinations.

### 4.7 The effects of different types of exercise, single intervention duration, and tDCS single stimulation duration on cognitive function in elderly individuals

Our meta-regression analysis showed that the single stimulation time of tDCS has a significant positive regulatory effect on cognitive function in elderly people, whereas the single exercise time and total intervention period show a significant negative regulatory effect. These findings are consistent with existing literature on neurophysiological mechanisms and clinical intervention studies, providing important evidence for optimizing combined intervention plans. The positive regulatory effect of tDCS single stimulation time indicates that longer single stimuli (approximately 15.6 min or more) are more conducive to inducing cognitive gains. This threshold is highly consistent with the basic mechanism of action of tDCS: Nitsche and Paulus confirmed that anodal tDCS requires at least 13 min to induce long-term potentiation-like effects through NMDA receptor-dependent synaptic plasticity mechanisms, and the subsequent effects can last for 30 to 120 min (Nitsche and Paulus, 2000; Nitsche and Paulus, 2001). In the original studies included in this analysis, Xu et al. (2023) used anodal tDCS over the dorsolateral prefrontal cortex for 20 min at 2 mA per session, combined with Tai Chi or brisk walking intervention for 12 weeks, which significantly improved overall cognitive, memory, and attention functions in patients with mild cognitive impairment (Ying et al., 2024). Clark et al. (2021) also used 20-min frontal tDCS combined with walking rehabilitation to achieve moderate-to-large improvements in executive function. In contrast, interventions with excessively short stimulation times (e.g., 10 min) often fail to produce consistent cognitive benefits (Horvath et al., 2015).

The negative moderating effect of the total intervention period (weeks) suggests that indefinite extension of the intervention may lead to diminishing returns. Several reasons may explain this phenomenon. First, cognitive improvement typically progresses most rapidly during the first 12–16 weeks of intervention before entering a plateau. Sanders et al. (2019) and Talar et al. (2022) noted that interventions lasting longer than 24 weeks often yield smaller effects, which may be attributed to reduced compliance and motivation, or the natural progression of cognitive function in MCI/dementia populations. Second, the type of exercise is crucial: interventions with monotonous content (e.g., simple walking) may lose efficacy over time, whereas programs incorporating progressive cognitive challenges (e.g., Tai Chi or dual-task training) may sustain benefits over extended periods. Both Xu et al. (2023) and Liao et al. (2021) reported that a 12-week Tai Chi intervention combined with tDCS significantly improves cognition (Ying et al., 2024; Liao et al., 2021); however, longer cycles (e.g., 6 months) do not necessarily yield greater cognitive benefits without escalating cognitive difficulty. Excessive prolonged stimulation may induce steady-state downregulation of synaptic plasticity (Hurley and Machado, 2017). Therefore, this study suggests that the intervention period should be optimized to approximately 12–16 weeks rather than extended indefinitely.

The negative moderating effect of single exercise duration suggests that prolonged individual exercise sessions may attenuate cognitive improvement. This finding is consistent with the inverted U-shaped hypothesis of motor-cognitive interaction and the catecholamine theory (Terry McMorris and Hale, 2012). Moderate-intensity aerobic exercise lasting 20–30 min typically enhances executive function by increasing prefrontal oxygenation and dopamine/norepinephrine release (Steinberg et al., 2019). However, prolonged exercise (e.g., exceeding 45 min) may induce central fatigue, reduce frontal lobe activity, and increase neural noise, thereby impairing cognitive performance (Arne Dietrich and Audiffren, 2011; Kate Lambourne and Tomporowski, 2010). In the original studies included in this review, experiments using longer single sessions (e.g., 60 min of walking or power cycling) often reported smaller or statistically non-significant cognitive effects, whereas experiments using moderate-duration exercise of 30–40 min yielded better outcomes. For instance, Conceição et al. (2021) observed improvements in gait variability and processing speed in Parkinson’s disease patients using a 30-min aerobic power cart combined with tDCS. In contrast, some dementia studies found that single exercise sessions lasting over 50 min did not confer additional cognitive benefits (Talar et al., 2022). Therefore, the present study supports controlling the duration of a single exercise session to approximately 30–40 min to avoid excessive fatigue and maintain optimal prefrontal cortex activation.

## 5 Limitations

This study also has several limitations. In this paper, only 10 eligible studies were retrieved from Chinese and English databases. The limited number of empirical studies available for analysis restricts the explanatory power of the research results. Furthermore, implementing blinding in exercise interventions is challenging, and tDCS interventions involve multiple variable parameters (e.g., intensity, duration, stimulation site, and polarity), which complicates a unified interpretation of the “joint effect.” These factors collectively contribute to the high heterogeneity of the present findings, warranting cautious interpretation. Future research should expand the empirical evidence in this field, focusing on potential dose–response relationships between different exercise programs and tDCS parameters, including the specific effects of stimulus intensity, duration, location, polarity, and other variables.

## 6 Conclusion

Based on limited evidence, we found that a combined intervention of exercise and tDCS is a potentially effective means of improving cognitive function in older adults. However, the extent of improvement varies with the cognitive domain, baseline performance level, and intervention plan.

## Data Availability

The original contributions presented in the study are included in the article/supplementary material, further inquiries can be directed to the corresponding author.
